# Immunosuppressive microvesicles-mimetic derived from tolerant dendritic cells to target T-lymphocytes for inflammation diseases therapy

**DOI:** 10.1186/s12951-024-02470-z

**Published:** 2024-04-24

**Authors:** Minghao Lin, Siyun Lei, Yingqian Chai, Jianghua Xu, Youchao Wang, Chenghu Wu, Hongyi Jiang, Shanshan Yuan, Jilong Wang, Jie Lyu, Mingqin Lu, Junjie Deng

**Affiliations:** 1 Joint Centre of Translational Medicine, The First Affiliated Hospital of Wenzhou Medical University, Wenzhou, 325000 Zhejiang China; 2 Joint Centre of Translational Medicine, Wenzhou Institute, University of Chinese Academy of Sciences, Wenzhou, 325000 Zhejiang China; 3 Zhejiang Engineering Research Center for Tissue Repair Materials, Wenzhou Institute, University of Chinese Academy of Sciences, Wenzhou, 325000 Zhejiang China; 4 Wenzhou Traditional Chinese Medicine Hospital, Wenzhou, 325000 China; 5 Chimie ParisTech, Institute of Chemistry for Life and Health Sciences, Laboratory for Inorganic Chemical Biology, PSL University, CNRS, Paris, 75005 France

**Keywords:** Immunosuppression, Microvesicle, Tolerant dendritic cell, Targeting, T-lymphocyte, Inflammation disease

## Abstract

The utilization of extracellular vesicles (EV) in immunotherapy, aiming at suppressing peripheral immune cells responsible for inflammation, has demonstrated significant efficacy in treating various inflammatory diseases. However, the clinical application of EV has faced challenges due to their inadequate targeting ability. In addition, most of the circulating EV would be cleared by the liver, resulting in a short biological half-life after systemic administration. Inspired by the natural microvesicles (MV, as a subset of large size EV) are originated and shed from the plasma membrane, we developed the immunosuppressive MV-mimetic (MVM) from endotoxin tolerant dendritic cells (DC) by a straightforward and effective extrusion approach, in which DC surface proteins were inherited for providing the homing ability to the spleen, while αCD3 antibodies were conjugated to the MVM membranes for specific targeting of T cells. The engineered MVM carried a large number of bioactive cargos from the parental cells, which exhibited a remarkable ability to promote the induction of regulatory T cells (Treg) and polarization of anti-inflammatory M2 macrophages. Mechanistically, the elevated Treg level by MVM was mediated due to the upregulation of miR-155-3p. Furthermore, it was observed that systemic and local immunosuppression was induced by MVM in models of sepsis and rheumatoid arthritis through the improvement of Treg and M2 macrophages. These findings reveal a promising cell-free strategy for managing inflammatory responses to infections or tissue injury, thereby maintaining immune homeostasis.

**Supplementary Information:**

The online version contains supplementary material available at 10.1186/s12951-024-02470-z.

## Graphical Abstract

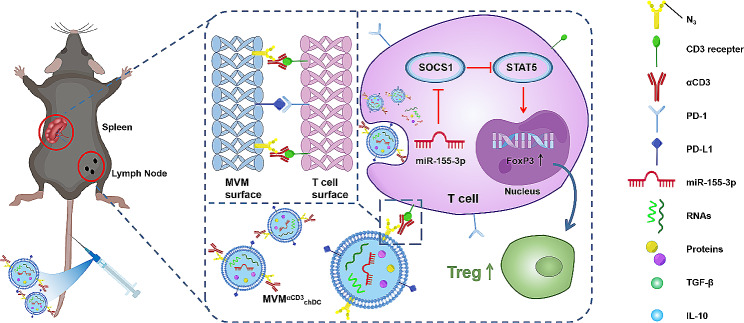

## Background

Acute or chronic inflammation diseases, such as sepsis and rheumatic arthritis, have become increasingly prevalent in clinical practices. The conditions are characterized by systemic dysregulation of inflammatory and excessive immune responses [1, 2]. Immunotherapy based on suppression of pro-inflammatory peripheral immune cells (neutrophils, macrophages and T lymphocytes) has been proven effective in various treatments of inflammatory diseases [3, 4]. Tolerant dendritic cells (DC) play a critical role in immune suppression therapy [5] through various mechanisms, including depletion of auto-reactive T cells [6], promotion of regulatory T cells (Treg) [7, 8] and expression of inhibitory molecules [9, 10]. For instance, endotoxin tolerant DC expressed low levels of costimulatory molecules and secrete immunosuppressive cytokines, thereby alleviating the peripheric immune response of asthmatic mice and sepsis-related liver injury model mice by increasing Treg and inhibiting pro-inflammatory macrophages [11, 12]. Although the ex vivo-generated tolerant DC have demonstrated feasibility and safety in the treatment of inflammatory diseases [13–16], but the immunomodulatory effect reported in clinical studies was not ideal due to poor engraftment and cell survival of the infused cells [17].

Compared to direct infusion of DC, DC-derived extracellular vesicles (EV, particularly exosomes) have emerged as a promising method for immunomodulation. These EV carry a variety of bioactive molecules transferred from the parental DC, including nucleic acid, proteins, RNAs and lipids [18, 19], which also have the notable ability to be stored at -80 °C for a long period and high biostability in circulation [20]. However, the application of DC-derived EV as a cell-free therapy encountered several limitations, such as low yield, lack of targeting, short blood circulation time and rapid clearance by the liver [21–23], resulting in frequent dosing of EV and poor immunosuppressive efficiency. Therefore, it is of significance to develop a scalable approach to prepare DC-derived EV with the ability to target T cells and improve immunosuppression. In contrast to small EV (mainly referred to as exosomes, typically < 150 nm in diameter) formed within cells, large EV (mainly referred to as microvesicles, MV, 200 nm to 2 μm in diameter) are more likely to play a crucial role in biology and disease [24], due to the fact that MV are formed and released from the plasma membrane and their larger size are capable of carrying more bioactive cargos, including signalling proteins directly from the plasma membrane and nucleic acids from the parental cells [25, 26]. As a result, DC-derived MV which inherited surface proteins from DC plasma membrane had a significant homing ability to immune organs (spleen and lymph node). Meanwhile, the genetic manipulation or chemical modification can also facilitate the engineering of MV surfaces to improve their cell targeting. Overall, the engineering tolerant DC-derived MV with T cell targeting may enhance the immunosuppressive regulation of T cells in immune organs, while also improving anti-inflammatory activity in peripheral blood, providing a novel approach for the treatment of acute and chronic inflammatory diseases.

**Scheme 1:**
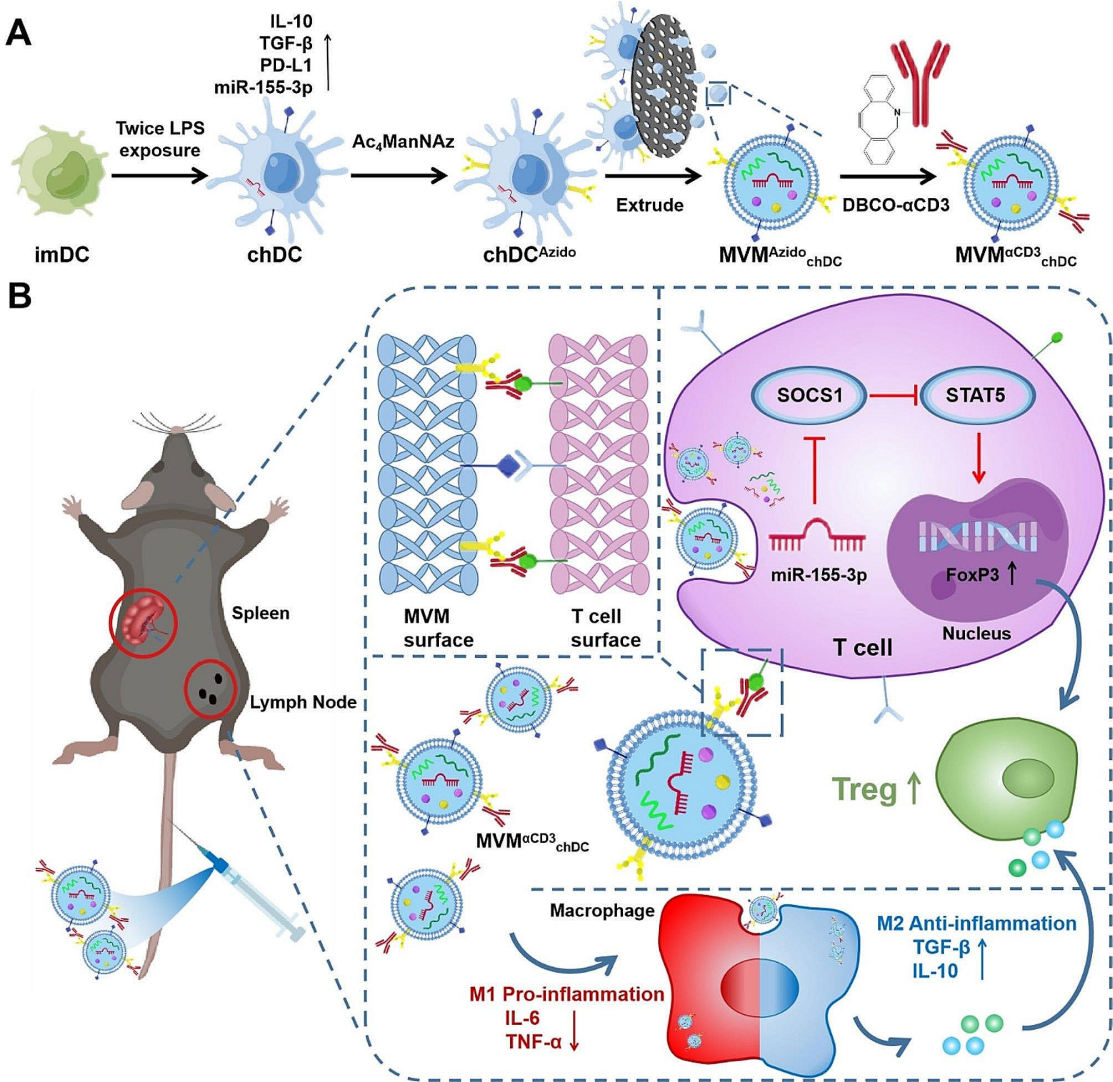
Schematic illustration of MVM^αCD3^_chDC_ preparation and the therapeutic mechanism. **A** Schematic of anti-CD3 antibodies-engineered chDC-derived microvesicle mimetic (MVM^αCD3^_chDC_) preparation. **B** MVM^αCD3^_chDC_ were highly efficiently distributed in spleen and LNs after intravenously injection through the homing ability of MV and the active targeting function of anti-CD3 antibodies on surface. MVM^αCD3^_chDC_ upregulated the proportion of Treg by delivering microRNA-155-3p and other cargos and promoted immune tolerance in acute inflammatory model and autoimmune disease, which also inhibited the pro-inflammatory M1 macrophages and improved anti-inflammatory M2 macrophages

To overcome the limitation of low productivity of natural EV secretion in clinical application [21], a prevailing approach is the serial extrusion of cells through microporous filters to generate exosome-mimetic (EM) with similar size and composition to those of natural exosomes [27]. However, the studies of immunosuppressive extracellular vesicles are mainly focused on engineering exosomes [28–30]. The utilization of micovesicle mimetic to induce immunosuppression for treating inflammatory disease has not been reported to date, and their potential in this context remains poorly understood. Herein, with this in mind, we developed immunosuppressive MV-mimetic (MVM) from endotoxin tolerant DC by a simple and efficient extrusion approach with high yield, in which DC surface proteins were retained and αCD3 antibody was conjugated on the MVM membranes (Scheme 1). Engineered MVM displayed a significant homing ability to spleen and improved T cells targeting ability compared with EM. Notably, MVM possessed more bioactive cargos derived from parental cells and exhibited a remarkable ability to induce Treg and anti-inflammatory macrophage. Mechanistically, the upregulation of Treg in both MVM and their parental DC was found to be associated with a significant increase in microRNA-155 levels. Meanwhile, MVM had the ability to induce rapid and effective immunosuppression in both the sepsis model and the rheumatoid arthritis (RA) model. To the best of our knowledge, this work provides a novel cell-free immunosuppression strategy for the treatment of acute and chronic inflammation diseases.

## Results

### Preparation and characterization of MVM^αCD3^_chDC_

**Fig. 1:**
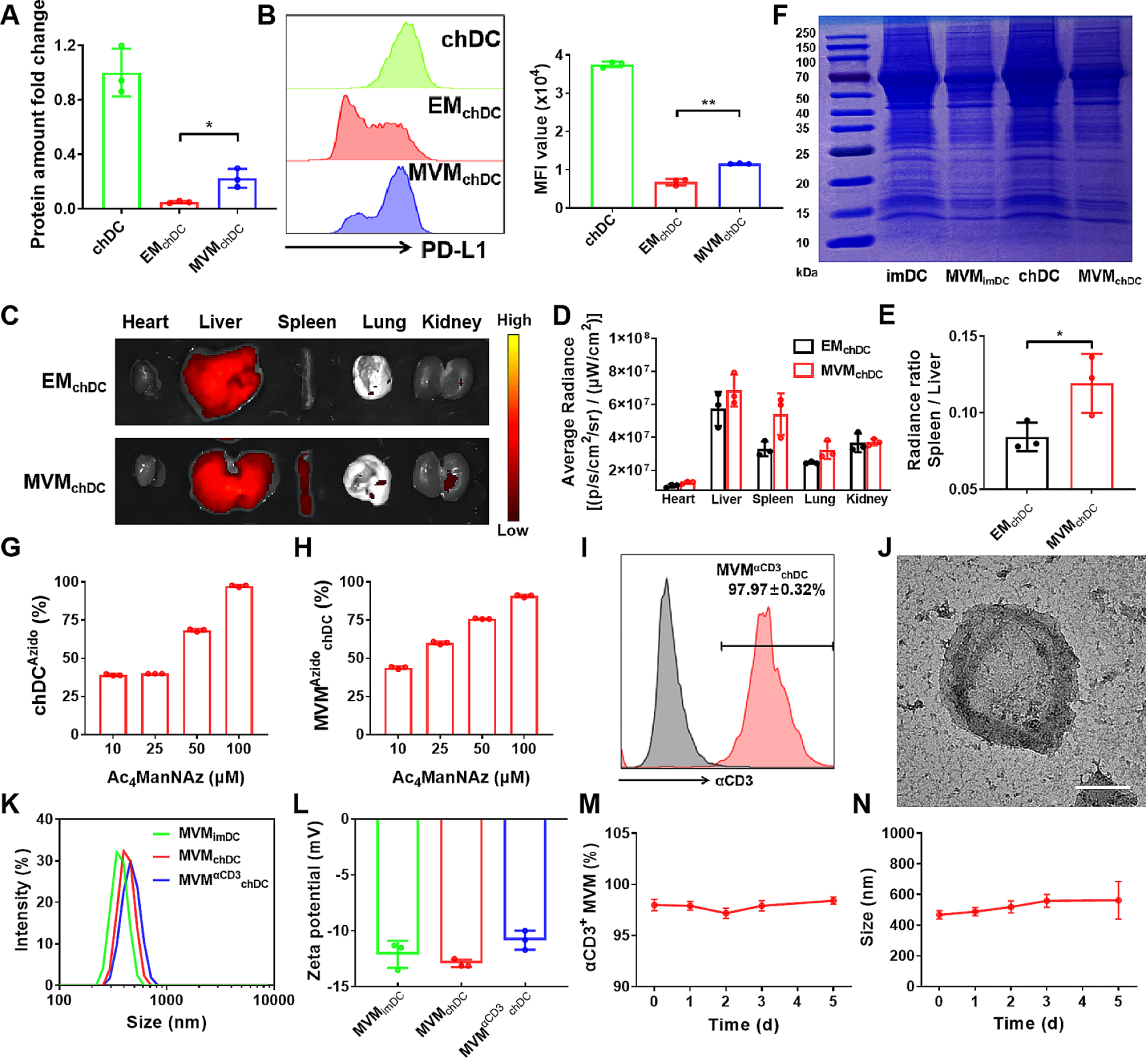
Characterization of MVM^αCD3^_chDC_. **A** The protein amount fold change of EM_chDC_ and MVM_chDC_ from a constant number of chDC. **B** The PD-L1 mean fluorescence intensity of chDC, EM_chDC_ and MVM_chDC_ were determined by flow cytometry analysis. **C**Ex vivo tissue distribution of EM_chDC_ and MVM_chDC_ at 24 h after tail vein injection. EM_chDC_ and MVM_chDC_ were labeled using DiD fluorescence. **D** Quantitative analysis of EM_chDC_ and MVM_chDC_ in heart, liver, spleen, lung, and kidney. **E** The radiance ratio of spleen to liver for EM_chDC_ and MVM_chDC_ in mice. **F** Image of sodium dodecyl sulfate polyacrylamide gel electrophoresis (SDS-PAGE) protein analysis. **G-H** Percentage of azido-labeled chDC and MVM_chDC_ after different concentration of Ac_4_ManNAz treatments. chDC were treated with different concentrations of Ac_4_ManNAz for 3 days. **I** Percentage of αCD3-positive MVM^Azido^_chDC_ after 2 h co-culture with DBCO-αCD3. Grey indicated control fluorescence in MVM^Azido^_chDC_ that were not incubated with DBCO-αCD3. **J** TEM image of MVM^αCD3^_chDC_. Scale bar = 200 nm. **K-L** Hydrodynamic diameter and ζ-potential of MVM_imDC_, MVM_chDC_ and MVM^αCD3^_chDC_. **M-N** αCD3 antibodies stability and size stability of MVM^αCD3^_chDC_ in PBS with serum for 5 days. Data were presented as mean ± SD. Statistical analyses were performed by Student’s *t*-test. *N* = 3 per group. ns *P* > 0.05, *P < 0.05, and **P < 0.01

Bone marrow monocytes were isolated from the C57BL/6 mice and were then stimulated to obtain bone marrow-derived dendritic cells (BMDC) according to a reported method [31]. Flow cytometric analysis confirmed that more than 80% of cells expressed DC marker CD11c (Fig. S1A). BMDC as immature DC (imDC) were stimulated twice using lipopolysaccharide (LPS) with different concentrations to obtain challenge DC (chDC), which was reported as endotoxin tolerant DC [12, 32]. Compared to pro-inflammatory mature DC (mDC), chDC had an obviously decrease of TNF-α and IL-12 expression, and a significantly increase of IL-10 and TGF-β expression (Fig. S1B), suggesting that the endotoxin tolerant DC were successfully obtained. Additionally, chDC displayed imDC-like morphology, with less protrusions on their surface (Fig. S1C). To investigate the the expression of surface markers on DCs, the levels of CD80, CD86, MHC II and PD-L1 were detected using flow cytometry. As shown in Fig. S1D, chDC had lower expressions of CD80, CD86 and MHC II compared with mDC. Meanwhile chDC had the highest expression of PD-L1 than other groups. ImDC and chDC were chosen to prepare imDC-derived MVM (MVM_imDC_) and chDC-derived MVM (MVM_chDC_) using the extrusion method, respectively (Scheme 1), which had a similar size distribution with peak diameter of around 400 nm [MVM_imDC_: about 360 nm, the polydispersity index (PDI) of 0.21; MVM_chDC_: about 418 nm, PDI of 0.22 in Fig. 1K] and similar zeta potentials of around − 12 mV (MVM_imDC_: -12.1 ± 1.2 mV, MVM_chDC_ : -12.9 ± 0.4 mV, Fig. 1L). The chDC-derived EM (EM_chDC_) was also prepared using extrusion with progressively reduced pore size membrane (Fig. S2A), and EM_chDC_ had the dynamic size of 175.5 nm (PDI: 0.28) and zeta potentials of -13.7 ± 1.1 mV (Fig. S2B). Comparing with EM_chDC_, MVM_chDC_ displayed ∼ 4.6-fold increase of protein amount in terms of a constant number of chDC (Fig. 1A), indicating that MVs were capable of carrying more bioactive cargos than that of exosome with smaller size. Furthermore, PD-L1 was chosen as a specific surface protein to detect the difference of surfaces between EM_chDC_ and MVM_chDC_. The PD-L1 mean fluorescence intensity (MFI) of MVM_chDC_ was significantly higher than that of EM_chDC_ (Fig. 1B and S3), revealing that MVM_chDC_ could reserve more signalling proteins from chDC on the surface. We detected ex vivo tissue distribution of EM_chDC_ and MVM_chDC_ in C57BL/6 mice *via* tail vein injection. As shown in Fig. 1C-E, MVM_chDC_ was much higher accumulating in spleen than EM_chDC_. This may be due to the homing ability of MVM_chDC_ resulting from inheriting surface proteins from chDC, as well as the size effect related to the clearance of nanoparticles in the spleen [33]. Sodium dodecyl sulfate-polyacrylamide gel electrophoresis (SDS-PAGE) was performed to compare the protein compositions of DC and DC-derived MVM. The highly similar stripes between cells and MVM further manifested that the majority of cellular proteins were reserved in MVM using our extrusion method (Fig. 1F).

To prepare azido groups modified MVM_chDC_, azido groups modified chDC were firstly prepared through chDC co-culture with Ac_4_ManNAz that had been commonly used as an unnatural azido-sugar to label azido groups on cell surface glycoproteins *via* metabolic pathway [34]. To confirm whether azido groups labeled chDC, the cells were incubated with DBCO-Cy5 probe at 37 °C and then washed by PBS. As shown in Fig. 1G and S4, the percentage of Cy5-positive chDC increased with the concentration of Ac_4_ManNAz, and ∼ 95% of chDC were Cy5-positive through the incubation of 100 µM Ac_4_ManNAz for 3 days, indicating a high labelling efficiency of azido groups on the surface of chDC. Then, we prepared azido groups labeled MVM_chDC_ (MVM^Azido^_chDC_) through the extrusion method and the percentage of modified azido groups on MVM^Azido^_chDC_ was evaluated using DBCO-Cy5 probe. Similarly, the percentage of Cy5-positive MVM_chDC_ increased with the concentration of Ac_4_ManNAz (Fig. 1H and S5). These data indicated that our extrusion approach could effectively reserve the azido groups from cells plasma membrane to MVM surface, further mimicking natural MV that were released directly from the plasma membrane and could intactly remain cell surface receptors and signalling proteins.

To prepare αCD3 modified MVM, DBCO-αCD3 antibody was synthesized and then incubated with azido-labeled chDC for 2 h. To confirm the conjugation of αCD3 antibodies on MVM surface *via* click reaction, MVM^Azido^_chDC_ were incubated with DBCO-αCD3 and then stained with secondary antibody for flow cytometry analysis. Figure 1I revealed the generation of near 98% of αCD3-positive MVM^ɑCD3^_chDC_, suggesting the success in conjugating αCD3 antibodies. Transmission electron microscopy (TEM) imaging displayed MVM^ɑCD3^_chDC_ with spherical and uniform morphology (Fig. 1J). Dynamic light scattering (DLS) measurement demonstrated that MVM^ɑCD3^_chDC_ had a narrow size distribution with peak diameter of around 456 nm (PDI: 0.16), which was consistent with the outcomes of TEM analysis (Fig. 1K). And the zeta potentials of MVM^αCD3^_chDC_ was − 10.8 ± 0.9 mV (Fig. 1L), which was similar to that of natural MV (around − 20 mV) [35]. To investigate the stability of MVM^αCD3^_chDC_, MVM^αCD3^_chDC_ was suspended in PBS with 10% FBS and it had excellent stability both in αCD3 antibodies conjugation and vesicle size (Fig. 1M, N and S6). The in vitro cytotoxicity of DC-derived MVM in murine primary splenic lymphocytes were evaluated using CCK-8 assays. All of MVM_imDC_, MVM_chDC_ and MVM^αCD3^_chDC_ represented reliable biocompatibility (Fig. S7). Collectively, these results indicated that MVM^αCD3^_chDC_ with semblable membrane surface and comparable protein composition from chDC could be produced by a facile extrusion method. Furthermore, we could modify αCD3 antibodies on the surface of MVM efficiently *via* click reaction.

### T cell-targeting ability of MVM^αCD3^_chDC_ in vitroand in vivo

**Fig. 2:**
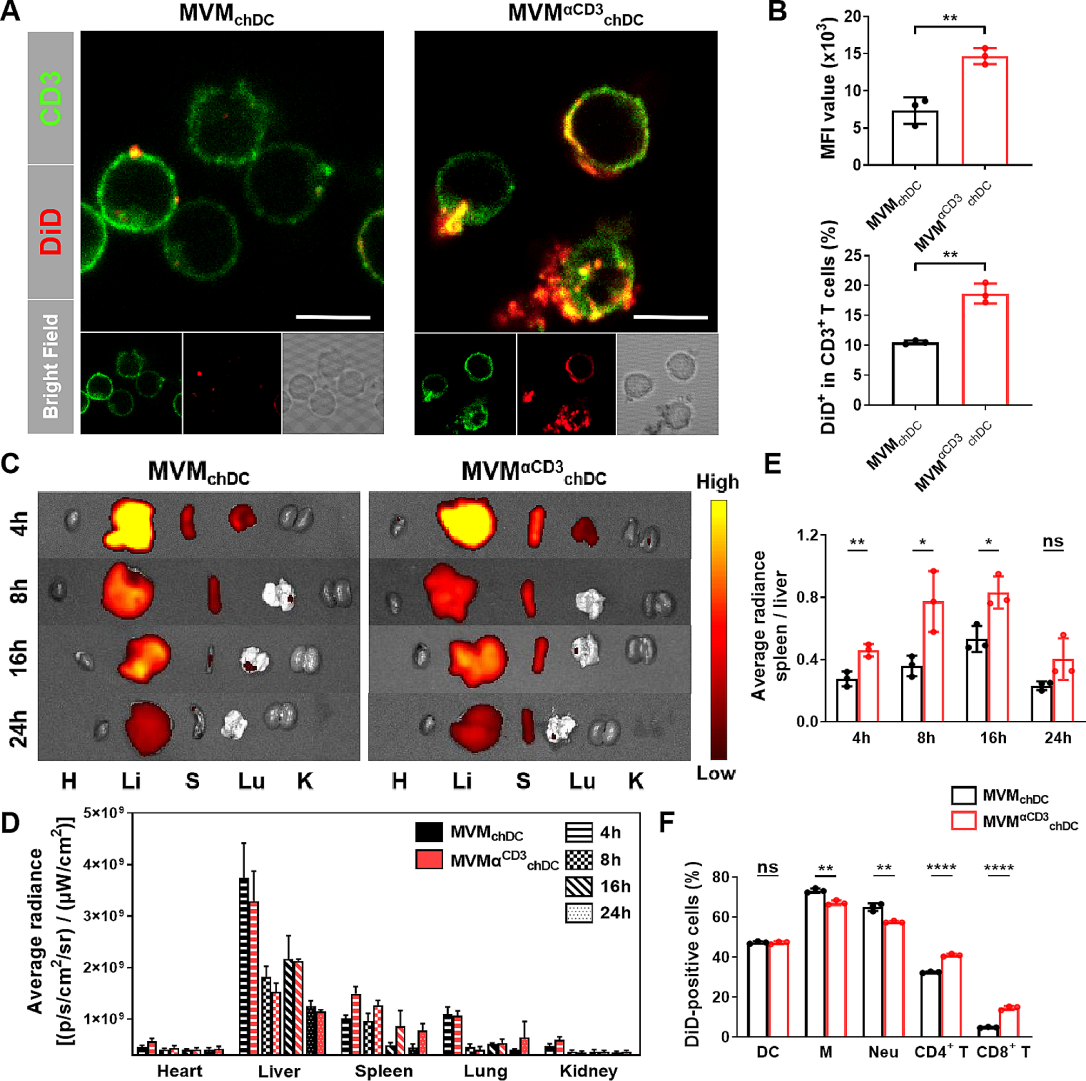
T cell-targeting capability and biodistribution of MVM^αCD3^_chDC_. **A** Intracellular uptake of MVM_chDC_ and MVM^αCD3^_chDC_ in T cells after 8 h incubation. MVM_chDC_ and MVM^αCD3^_chDC_ were labeled using DiD (red). T cell membrane was stained with FITC secondary antibody (green). Scale bar = 5 μm. **B** The corresponding intracellular fluorescence signals were determined by flow cytometry analysis. **C**Ex vivo tissue distribution of MVM_chDC_ and MVM^αCD3^_chDC_. H : heart; Li : liver; S : spleen; Lu : lung; K : kidney. Mice were intravenously injected with MVM_chDC_ and MVM^αCD3^_chDC_ at different time points. MVM_chDC_ and MVM^αCD3^_chDC_ were labeled using DiD fluorescence. **D** Quantitative analysis of MVM_chDC_ and MVM^αCD3^_chDC_ in heart, liver, spleen, lung, and kidney at different time points. **E** The average radiance ratio of spleen to liver for MVM_chDC_ and MVM^αCD3^_chDC_ at different time points. **F** Uptake of DiD labeled MVM_chDC_ and MVM^αCD3^_chDC_ by myeloid and lymphoid cells in spleen at 24 h. Data were presented as mean ± SD. Statistical analyses were performed by Student’s *t*-test. *N* = 3 per group. ns *P* > 0.05, *P < 0.05, **P < 0.01, ***P < 0.001, and ****P < 0.0001

αCD3 antibody was selected as a surface engineering targeting moiety in this work because of its specific binding affinity for the CD3 receptor, which is highly expressed on T cell populations [34]. To investigate whether the T cell-targeting ability of MVM^αCD3^_chDC_ can be further strengthened, splenic lymphocytes were treated by DiD-labeled MVM_chDC_ and MVM^αCD3^_chDC_ for 8 h. Confocal microscope imaging demonstrated that αCD3 antibodies modification enhanced MVM^αCD3^_chDC_ internalization by T lymphocytes (Fig. 2A and S8). Flow cytometry analysis showed that the internalization of MVM^αCD3^_chDC_ by T lymphocytes was nearly 2 times higher than that of MVM_chDC_ (Fig. 2B and S9).

To study the targeting efficiency of MVM^αCD3^_chDC_in vivo, mice were intravenously injected with DiD-labeled MVM_chDC_ and MVM^αCD3^_chDC_, and the major organs were then harvested and examined at different time points (Fig. 2C). Both MVM_chDC_ and MVM^αCD3^_chDC_ demonstrated the highest accumulation in liver, consistent with a previous study that natural EV and EV mimetics were mainly cleared away from liver [23]. In addition, MVM_chDC_ without αCD3 antibodies modification also accumulated in spleen within the initial 8 h. This observation could be attributed to MVM_chDC_ inheriting the surface bioactive molecules from chDC plasma membrane, resulting in limited homing ability. Moreover, MVM^αCD3^_chDC_ exhibited a higher accumulation in the spleen compared to MVM_chDC_, with the peak reached at 4 h after injection (Fig. 2C, D). Accordingly, Fig. 2E showed that the average radiance ratio of spleen to liver was significantly elevated for MVM^αCD3^_chDC_, indicating that αCD3 antibodies conjugation obviously enhanced the splenic accumulation of MVM^αCD3^_chDC_. Furthermore, we detected the cell distributions of MVM^αCD3^_chDC_ in spleen and found more MVM^αCD3^_chDC_ were accumulated in T lymphocytes compared with MVM_chDC_ (Fig. 2F), which was consistent with the in vitro results. Meanwhile, we observed lymph nodes at different time points. As shown in Fig. S10, MVM^αCD3^_chDC_ exhibited a higher accumulation in the lymph nodes compared to MVM_chDC_. Taken together, these results demonstrated that the direct formations from the chDC plasma membrane provided MVM^αCD3^_chDC_ with significant homing ability in spleen, and the modification strategy of conjugating αCD3 antibodies enhanced the T cell-targeting capability of MVM^αCD3^_chDC_.

### Regulation of immune cells by MVM^αCD3^_chDC_ in vitro

**Fig. 3:**
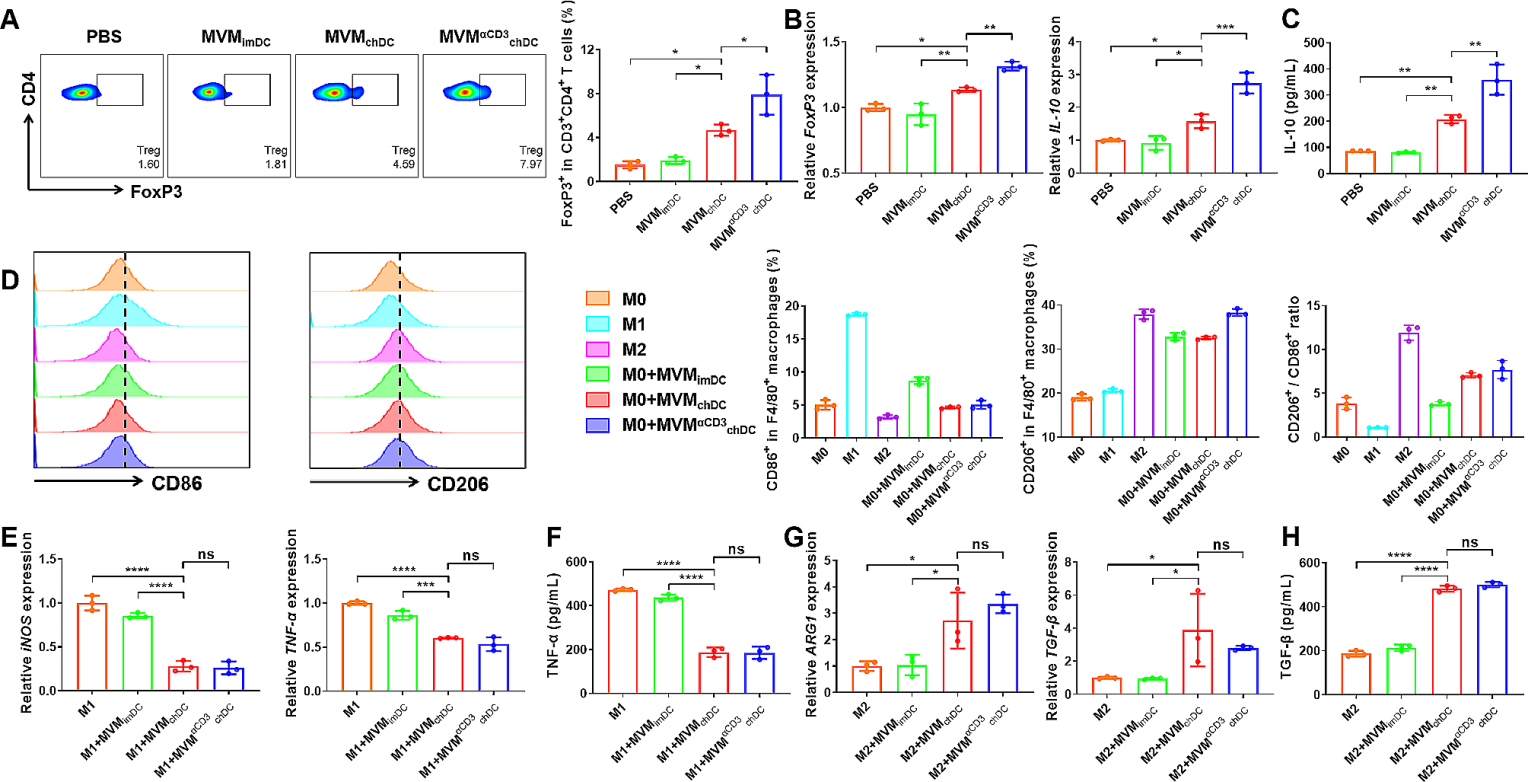
MVM^αCD3^_chDC_ promoted Treg polarization of T cells and M2 polarization of macrophages in vitro. **A** Percentage of the CD3^+^CD4^+^FoxP3^+^ Treg in CD3^+^CD4^+^ T cells using flow cytometry analysis. **B** Relative mRNA expression of FoxP3 and IL-10 of splenic lymphocytes after different treatments. **C** The concentration of IL-10 in supernatant of splenic lymphocytes after different treatments. **D** Percentage of F4/80^+^CD86^+^ M1 and F4/80^+^CD206^+^ M2 in F4/80^+^ macrophages and the corresponding M2/M1 ratio using flow cytometry analysis. **E** Relative mRNA expression of iNOS and TNF-α of M1 macrophage after different treatments. **F** The concentration of TNF-α in supernatant of M1 macrophages after different treatments. **G** Relative mRNA expression of ARG-1 and TGF-β of M2 macrophages after different treatments. **H** The concentration of TGF-β in supernatant of M2 macrophages after different treatments. Data were presented as mean ± SD. Statistical analyses were performed by One-way ANOVA with Tukey method. *N* = 3 per group. ns *P* > 0.05, *P < 0.05, **P < 0.01, ***P < 0.001, and ****P < 0.0001

To investigate whether MVM^αCD3^_chDC_ had similar effects on Treg as chDC, splenic lymphocytes were co-cultured with different MVMs for 72 h and were then examined using flow cytometry analysis. Compared to MVM_imDC,_ MVM_chDC_ increased the average level of CD3^+^CD4^+^FoxP3^+^ Treg in splenic lymphocytes from 1.9 ± 0.3% to 4.7 ± 0.5%, Moreover, MVM^αCD3^_chDC_ exhibited a further increase in the proportion to 7.9 ± 1.8% (Fig. 3A and S11). It may be contributed to MVM^αCD3^_chDC_ had improved T lymphocytes internalization. Meanwhile, there was no significant difference in Treg induction between MVM_imDC_ and MVM^αCD3^_imDC_, further confirming that the important role of MVM_chDC_ in Treg induction (Fig. S12). The representative transcription factor (Foxp3) and cytokine (IL-10) of Treg were also confirmed by qPCR measurement (Fig. 3B). MVM^αCD3^_chDC_ treatment showed the highest expression of Foxp3 and IL-10 than other treatments. As expected, the secretion of IL-10 in cell supernatant was measured using ELISA. As shown in Fig. 3C, MVM^αCD3^_chDC_ treatment had the highest release of IL-10, indicating an effectively enhancement of Treg induction.

Furthermore, to investigate whether MVM affected the polarization of macrophage phenotype, we co-cultured different MVMs with M0 macrophages [bone marrow derived macrophages (BMDM)] for 48 h. Confocal microscope imaging and flow cytometry analysis showed that macrophages as professional phagocytes had a strong phagocytosis of MVM^αCD3^_chDC_ (Fig. S13). As shown in Fig. 3D, compared to MVM_imDC_, both MVM_chDC_ and MVM^αCD3^_chDC_ exhibited stronger abilities to promote the polarization of anti-inflammatory F4/80^+^CD206^+^ M2 macrophage, while they did not induce pro-inflammatory F4/80^+^CD86^+^ M1 macrophage polarization. Further investigation was conducted on the impact of MVM on M1 and M2 macrophages. The inflammation related mRNA expressions (iNOS, TNF-α, and IL-6) and the anti-inflammation related mRNA expressions (ARG-1 and TGF-β) were determined using qPCR measurement. Compared with MVM_imDC_, both MVM_chDC_ and MVM^αCD3^_chDC_ significantly reduced iNOS, TNF-α, and IL-6 expressions in M1 macrophages (Fig. 3E and S14A), and obviously increased ARG-1 and TGF-β expressions in M2 macrophages (Fig. 3G). ELISA analysis further confirmed the effective downregulation of TNF-α and IL-6 production in M1 macrophages by MVM_chDC_ and MVM^αCD3^_chDC_ (Fig. 3F and S14B), while upregulating the secretion of TGF-β in M2 macrophages (Fig. 3H). Moreover, no difference was found between the MVM_chDC_ and MVM^αCD3^_chDC_ for macrophage regulation, indicating that the modification with αCD3 antibodies did not have additional effects on macrophages. We also prepared purified chDC membranes (membrane_chDC_) to identify the major anti-inflammatory constituents in MVM_chDC_. As shown in Fig. S15, there was no significant difference between PBS and membrane_chDC_ treatment groups in Treg induction and macrophage polarization, indicating that the immunosuppressive effects of MVM_chDC_ were primarily contributed to its internal components, rather than surface molecules on MVM_chDC_. Overall, these results suggested that MVM^αCD3^_chDC_ could exert enhanced immunosuppressive regulatory effects through innate or adaptive immune cells responses, including improved Treg induction, M2 macrophage polarization and anti-inflammatory cytokines secretion.

### The molecular mechanism of the induction of Treg by MVM_chDC_

**Fig. 4:**
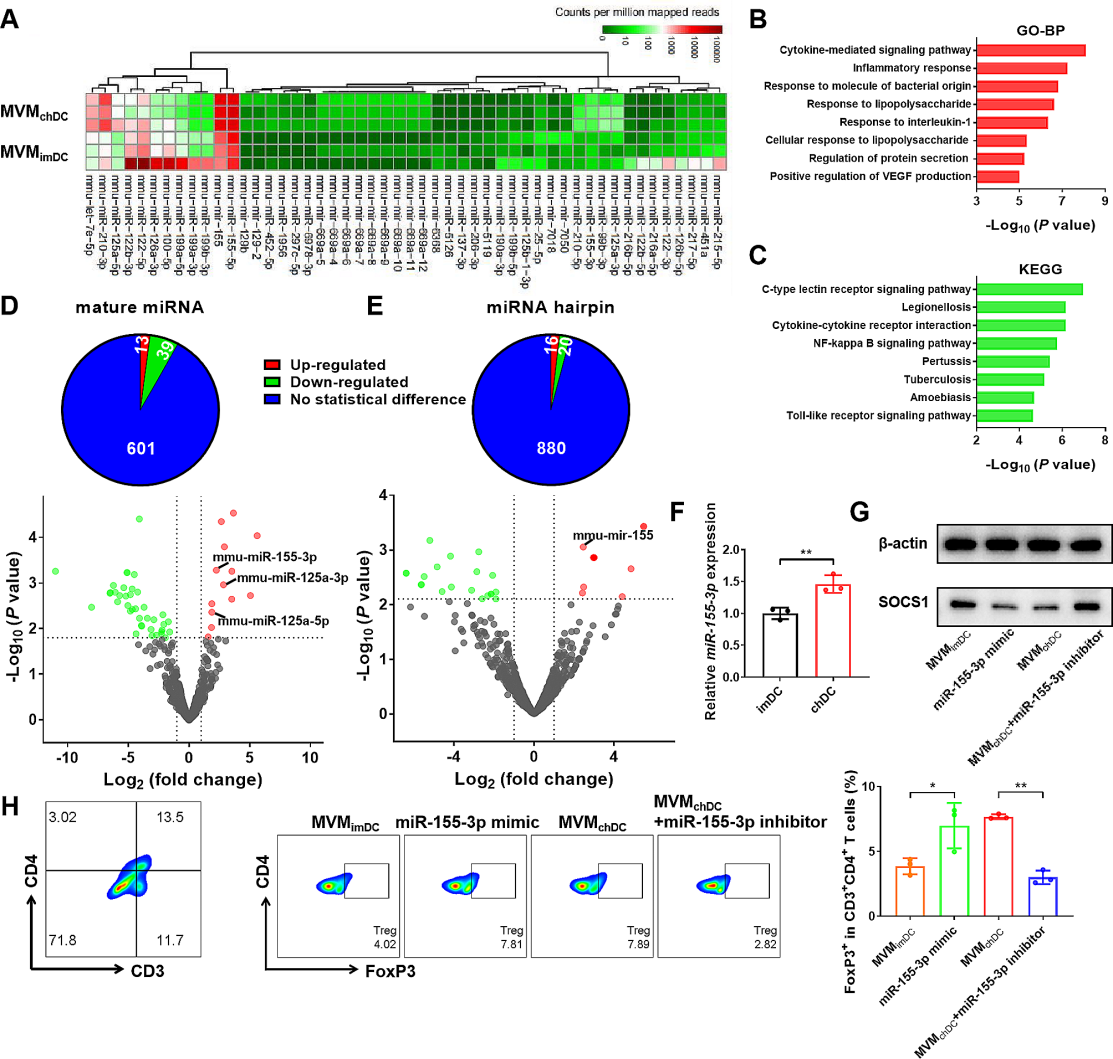
MVM_chDC_ promoted the proportion of Treg through upregulated miR-155-3p. **A** Differentially expressed microRNA haipin (mir) and mature microRNA (miR) between MVM_imDC_ and MVM_chDC_ were exhibited *via* a heat map. **B** Enriched terms of biological process of the upregulated genes by GO analysis. **C** Enriched terms of KEGG pathway analysis of the up-regulated genes. **D-E** Pie chart and volcano plot displaying the distribution of the down-regulated, up-regulated, and common miR or mir between MVM_imDC_ and MVM_chDC_. The red and green dots indicated the differentially expressed miRNAs. **F** Relative expression of miR-155-3p in imDC and chDC. **G** Expression levels of β-actin and SOCS1 protein in splenic lymphocytes after different treatments. **H** Percentage of the CD3^+^CD4^+^FoxP3^+^ Treg in CD3^+^CD4^+^ T cells after different treatments using flow cytometry analysis. Data were presented as mean ± SD. Statistical analyses were performed by Student’s *t*-test or One-way ANOVA with Tukey method. *N* = 3 per group. ns *P* > 0.05, *P < 0.05, **P < 0.01, ***P < 0.001, and ****P < 0.0001

To investigate the potential molecular mechanism underlying the immunosuppressive effects induced by MVM_chDC_, the next-generation sequencing (NGS) technology was used to evaluate the transcriptomic profiles of MVM_chDC_ and MVM_imDC_. Differential data analysis was conducted on the obtained sequencing data, revealing 52 mature miRNAs (prefix of mmu-miR-) and 36 miRNA hairpins (suffix of -5p or -3p) that exhibited either down- or up-regulated in MVM_chDC_*versus* MVM_imDC_ (FDR < 0.1). Next, we performed Gene Ontology (GO) and Kyoto Encyclopedia of Genes and Genomes (KEGG) term enrichment analyses to get insights into the functions of the upregulated genes in MVM_chDC_ compared to MVM_imDC_, which may imply a potential mechanism that conferred MVM_chDC_ upon the upregulation of the proportion of Treg (Fig. 4B, C, and S16). Biological process (BP) results indicated that these related genes were mainly involved in cytokine-mediated signaling pathway, inflammatory response, response to molecule of bacterial origin, response to lipopolysaccharide, response to interleukin-1, cellular response to lipopolysaccharide, regulation of protein secretion and positive regulation of vascular endothelial growth factor production, among others. Among the top 8 pathways of the KEGG enrichment data, cytokine-cytokine receptor interaction, C-type lectin receptor signaling pathway, NF-kappa B signaling pathway and Toll-like receptor signaling pathway may play a crucial part in upregulating the proportion of Treg. Among a total of 653 miRNAs, only 13 had significantly higher expression levels (FDR adjusted *P*-value < 0.2, fold-change > 1) in MVM_chDC_ compared to MVM_imDC_ (Fig. 4A, D), of which miR-155-3p, miR-125a-3p and miR-125a-5p were reported to be associated with Treg upregulation [36–39]. Among a total of 916 miRNA hairpins, only 16 had significantly higher expression levels in MVM_chDC_ compared with MVM_imDC_ (Fig. 4A, E). Intriguingly, we discovered that the expression of miR-155-3p and its hairpin (mir-155) were both significantly increased in MVM_chDC_. Then, we verified the enrichment of miR-155-3p in chDC using qPCR measurement and showed that miR-155-3p in chDC was significantly higher than that in imDC (Fig. 4F), which was consistent with sequencing data described above. Additionally, MVM_chDC_ also displayed a significant increase of miR-155-3p compared to EM_chDC_ with a constant number of chDC (Fig. S17), suggesting that MVM could carry more endogenous miRNAs inherited from cells. MiR-155 has been reported to enhance FoxP3 expression by inhibiting suppressor of cytokine signaling 1 (SOCS1) that is an important negative regulator of IL-2R/STAT5 signaling [40, 41]. To further confirm the role of miR-155-3p in Treg induction, miR-155-3p mimic and inhibitor were utilized to treat the splenic lymphocytes. To demonstrate the effect of miR-155-3p on SOCS1, the protein expressions of splenic lymphocytes with different treatments were analyzed. MiR-155-3p mimic and MVM_chDC_ reduced the expression of SOCS1, while miR-155-3p inhibitor could mitigate the effect of MVM_chDC_ (Fig. 4G). In addition, flow cytometry analysis indicated an increasing level of FoxP3 in miR-155-3p mimic and MVM_chDC_ treated cells compared with those in the control. Moreover, when MVM_chDC_ and miR-155-3p inhibitor were co-incubated with splenic lymphocytes, the proportion of Treg showed no significant difference compared to MVM_imDC_ treatment (Fig. 4H). Alternatively, it has been reported that EV could promoted the polarization of macrophages to M2 phenotype *via* triggering ETS homologous factor (EHF)-dependent activation of Akt/NF-κB signaling pathway by transferring miR-155 to macrophages [42]. Although miR-155 has been reported to be associated with Th17 differentiation [43, 44], our observations suggested that MVM_chDC_ had no significant impact on the proportion of Th17 cells in CD3^+^CD4^+^ T cells compared to the PBS group in vitro (Fig. S18). Consequently, these results revealed that miR-155-3p played a critical role in the immunosuppression of MVM_chDC,_ especially through the upregulation of Treg.

### MVM^αCD3^_chDC_ alleviates sepsis and improves survival rate

**Fig. 5:**
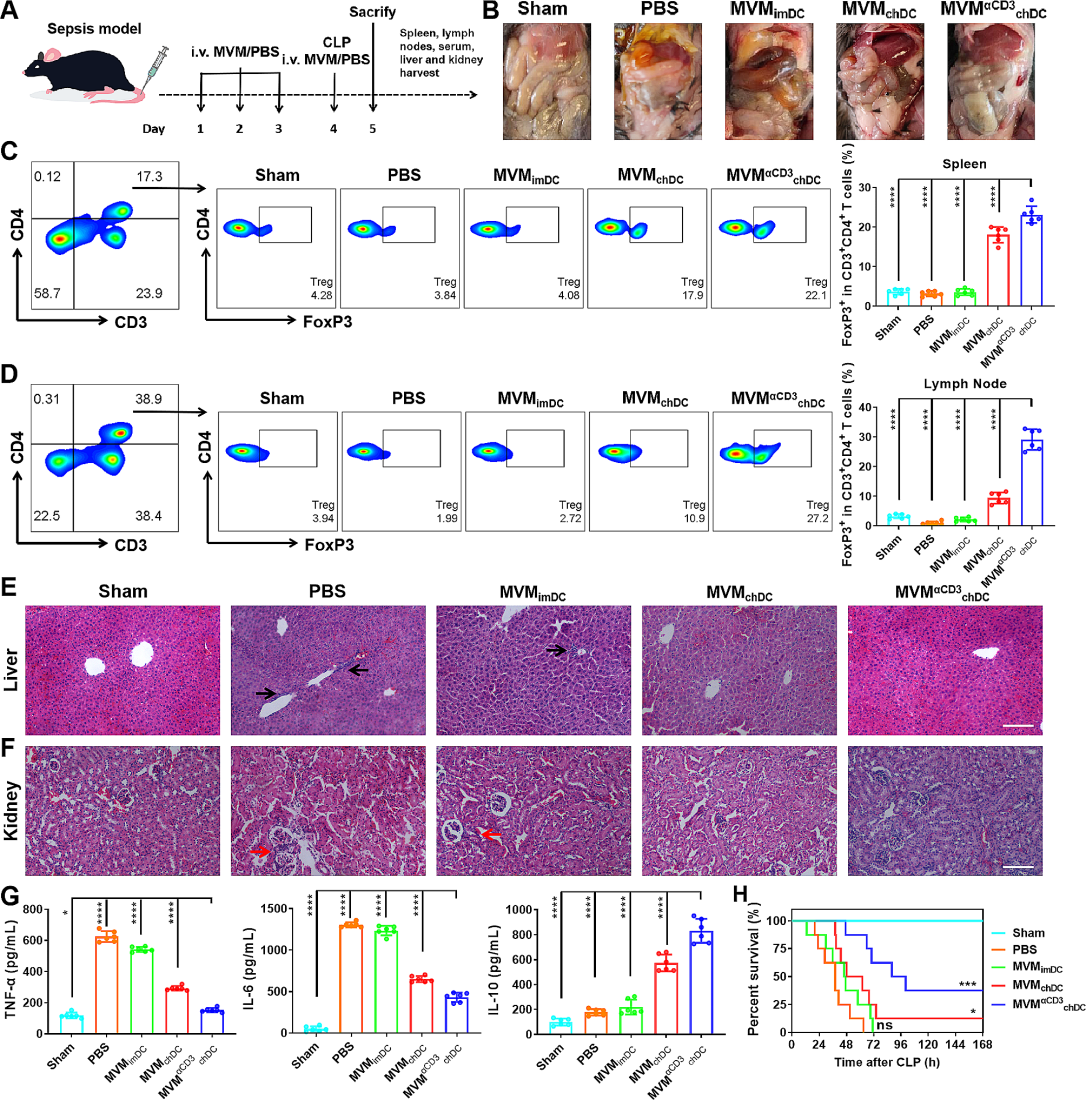
MVM^αCD3^_chDC_ exerted therapeutic effects in CLP-induced sepsis models. **A** Timeline of MVM^αCD3^_chDC_ treatment experiment in sepsis model. **B** Ventral view of mice from different treatment groups. **C-D** Percentage of CD3^+^CD4^+^FoxP3^+^ Treg in CD3^+^CD4^+^ T cells in spleen and lymph nodes in sepsis mice after different treatments using flow cytometry analysis. **E-F** H&E staining images of liver and kidney tissues after different treatments. Scale bar = 100 μm. Black and red arrows indicated invasion of inflammation. **G** The concentrations of TNF-α, IL-6, and IL-10 in the serum of mice after different treatments. **H** Survival curves of mice after different treatments. Mice with CLP-induced sepsis model continued to treatment every 24 h for 7 days instead of euthanizing at 24 h time point. Data were presented as mean ± SD. Statistical analyses were performed by One-way ANOVA with Tukey method. *N* = 6 per group. ns *P* > 0.05, *P < 0.05, **P < 0.01, ***P < 0.001, and ****P < 0.0001 compared with the MVM^αCD3^_chDC_ group. Statistical analyses in survival curves were performed by Log-rank tests, *N* = 8 per group. ns *P* > 0.05, *P < 0.05, **P < 0.01, and ***P < 0.001 compared with the PBS group

To explore whether the immunosuppressive potential of MVM^αCD3^_chDC_ is protective in acute inflammatory diseases, a murine sepsis model was treated with different MVMs by daily tail vein injections (from day 1 to day 3), and PBS treatment as a control (Fig. 5A). The cecal ligation and perforation (CLP) model was employed as one of the gold standards for sepsis research, triggering polymicrobial peritonitis, which ultimately led to septic shock. We established the sepsis *via* CLP at Day 4 and euthanized mice after 24 h. In both of PBS and MVM_imDC_ treatment groups, swelling and congestion were observed in the intestines of the mice, with purulent inflammation and a large amount of bloody turbid effusion produced in the abdominal cavity (Fig. 5B). MVM^αCD3^_chDC_ treatment group exhibited fewer purulent lesions in the abdominal cavity compared with other groups. Flow cytometry analysis demonstrated that MVM_chDC_ treatment increased the percentage of CD3^+^CD4^+^FoxP3^+^ Treg in spleen from 3.2 ± 0.7% to 18.1 ± 2.0%, and in lymph nodes from 1.0 ± 0.5% to 9.4 ± 1.9% compared with PBS treatment (Fig. 5C, D). When modifying αCD3 antibodies to obtain MVM^αCD3^_chDC,_ the percentage of population of CD3^+^CD4^+^FoxP3^+^ Treg further increased to 23.2 ± 2.1% and 29.2 ± 3.5% in spleen and lymph nodes, respectively, which contribute to more accumulation in spleen and internalization of MVM^αCD3^_chDC_ in T cells. Multiple organ dysfunction syndrome (MODS), a frequent and serious complication of sepsis, is associated with high mortality rates [45]. To study the therapeutic potential of MVM against CLP-induced MODS, the liver and kidney of mice were investigated *via* histological examination. Hematoxylin and eosin (H&E)-stained sections revealed that the septic mice treated with PBS or MVM_imDC_ had multiple diffuse coagulations in the liver and glomerular swelling with inflammatory cells infiltration in the kidney (Fig. 5E). However, MVM^αCD3^_chDC_ treatment considerably attenuated liver and kidney damage and showed less infiltration of inflammatory cells (Fig. 5F), similar as sham group. The immunofluorescence staining of M1 (CD86) and M2 (CD206) macrophages in liver of mice further confirmed that MVM^αCD3^_chDC_ promoted M2 macrophages and inhibited M1 macrophages (Fig. S19). Cytokine storm is an important characteristic of sepsis, which representing an auto-amplifying cytokine production [46]. The concentrations of key cytokines in the serum of mice were determined using ELISA measurement. Comparing to other groups, MVM^αCD3^_chDC_ treatment showed significantly reduced TNF-α and IL-6 levels in the serum (Fig. 5G). In contrast, a sharply up-regulated level of IL-10 in the serum that could be secreted by induced Treg and M2 macrophages was observed after MVM^αCD3^_chDC_ treatment. As expected, MVM^αCD3^_chDC_ treatment had the highest survival rate of 37.5% for 7 days, whereas the effect of MVM_imDC_ was relatively insignificant (Fig. 5H). These results indicated that MVM^αCD3^_chDC_ efficiently protected mice against sepsis through the induction of Treg and M2 macrophages.

### MVM^αCD3^_chDC_ ameliorates joint injury and inflammatory response in CIA model

**Fig. 6:**
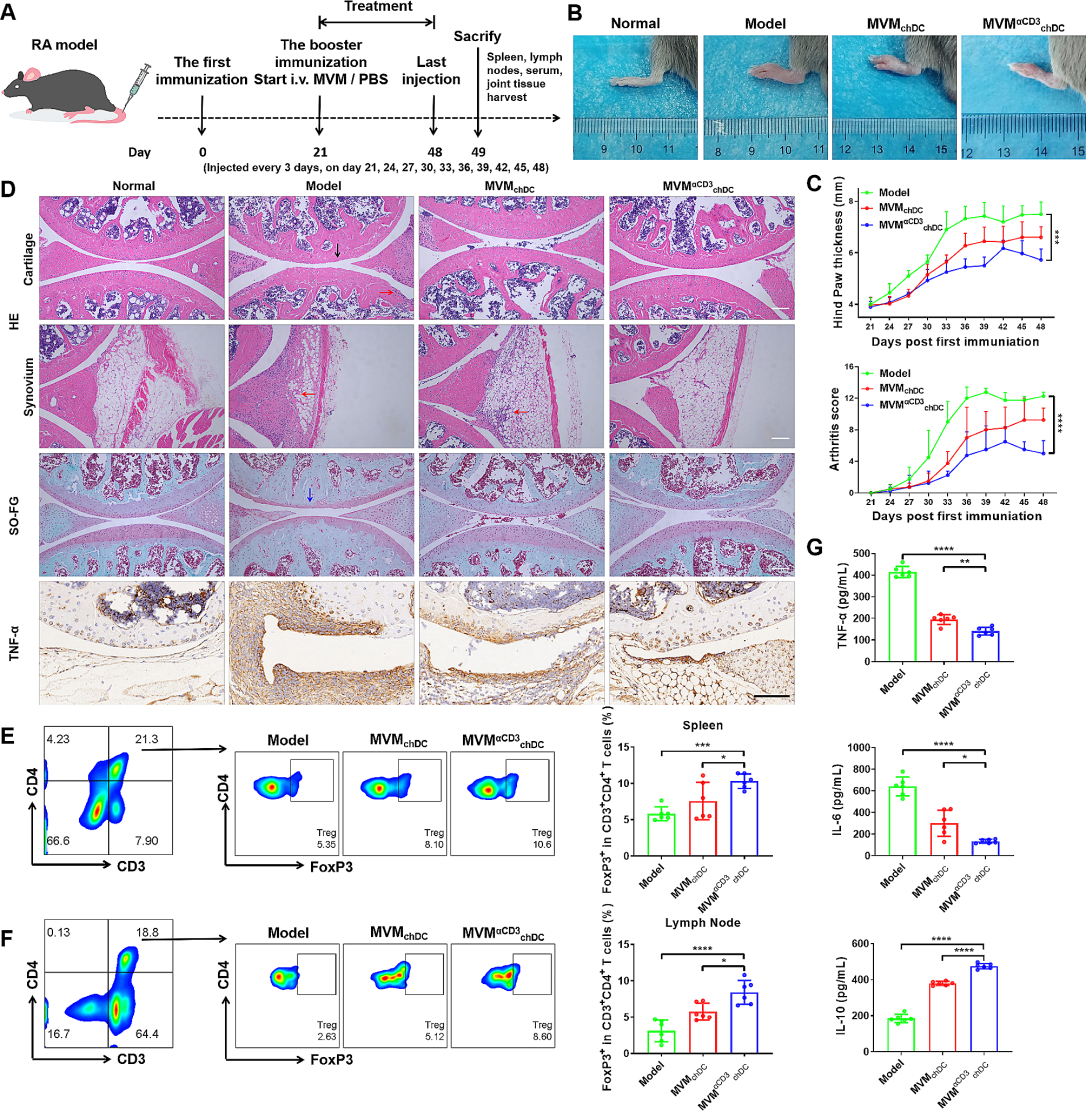
The effect of MVM^αCD3^_chDC_ on joint injury and inflammatory response in the CIA model. **A** Timeline of MVM^αCD3^_chDC_ treatment experiment in CIA model. **B** Macroscopic images of hind paws in mice after different treatments. **C** The change of hind paw thickness and arthritis scores in mice after different treatments. **D** H&E, SO-FG, and immunohistochemical staining images of histological sections in joints after different treatments. Scale bar = 100 μm. Black arrows indicated bone destruction and narrow joint space. Red arrows indicated invasion of inflammation. Blue arrows indicated loss of proteoglycan. **E-F** Percentage of the CD3^+^CD4^+^FoxP3^+^ Treg in CD3^+^CD4^+^ T cells in spleen and lymph nodes in CIA mice after different treatments using flow cytometry analysis. **G** The concentration of TNF-α, IL-6, and IL-10 in the serum of mice after different treatments. Data were presented as mean ± SD. Statistical analyses were performed by One-way ANOVA with Tukey method. *N* = 6 per group. ns *P* > 0.05, *P < 0.05, **P < 0.01, ***P < 0.001, and ****P < 0.0001

In order to assess the potential impact of MVM^αCD3^_chDC_ on autoimmune diseases associated with chronic inflammation, a collagen-induced arthritis (CIA) model with respect to rheumatoid arthritis process was developed and was treated every 3 days according to the schedule (Fig. 6A). Due to the lack of significant differences observed between PBS and MVM_imDC_ treatments in the previous experiments (data not shown), subsequent experiments did not include MVM_imDC_ treatment. Model group with PBS treatment displayed severe inflammation in the joints, while the paw thickness and arthritis score swelling obviously decreased after MVM^αCD3^_chDC_ treatment (Fig. 6B, C). RA is characterized by sustained synovitis, progressive cartilage and bone destruction [47]. In order to validate pathological states of RA at the end of treatment, histological analysis of the knee joint sections was performed (Fig. 6D). H&E staining images demonstrated the articular cavity surface damage, extensive inflammatory cells infiltration and obvious formation of pannus of mice in model group. However, the treatment with MVM^αCD3^_chDC_ exhibited articular cavity surfaces that were near normal, characterized by a clear interface and minimal infiltration of cells. In addition, safranin O-fast green (SO-FG) staining revealed a noticeable loss of proteoglycan in the model group, indicating severe cartilage damage. Remarkably, MVM^αCD3^_chDC_ treatment exhibited the best cartilage preservation except for the normal group. The anti-inflammatory effect of MVM^αCD3^_chDC_ was investigated through the histological examination of the proinflammatory cytokine expression. The expression of TNF-α in the model group was significantly elevated compared with the normal group, indicating the central involvement in the pathogenesis of RA. In MVM_chDC_ treatment, the expression of TNF-α was obviously decreased compared with PBS treatment, which was in accordance with the reduced synovial inflammation and cartilage erosion in above observations, while the expression in MVM^αCD3^_chDC_ group was further decreased. The therapeutic strategy that aims to achieve the immune homeostasis in spleen and lymph nodes by inducing Treg has great potential in RA therapy [48]. After MVM^αCD3^_chDC_ treatment, the percentage of CD3^+^CD4^+^FoxP3^+^ Treg were markedly increased in spleen and lymph nodes (Fig. 6E, F), while Treg-related cytokine (IL-10) was increased and pro-inflammatory cytokines (TNF-α and IL-6) were decreased in the serum of mice, respectively (Fig. 6G). To further evaluate the toxic and side effects of MVM treatments, major organs of treated mice were collected. No obvious pathological change was found during all groups, suggesting the biosafety of MVM (Fig. S20). Similarly, our results indicated that there is no significant difference in CD3^+^CD4^+^IL-17 A^+^ Th17 cells between MVM and model group, while Th17/Treg ratio showed significant differences (Fig. S21). Taken together, these results suggested that MVM^αCD3^_chDC_ could reduce inflammation in CIA by promoting Treg expansion and maintaining the immune homeostasis.

## Discussion

Acute or chronic inflammation diseases such as sepsis or RA, are mainly caused by the inflammatory disorder associated with the innate and adaptive systems [1, 2]. It is well known that Treg as an important immnosuppressive lymphocyte can exert their suppressive function both at the tissue site of inflammation and in secondary lymphoid tissues, mediating the anti-inflammatory response by secreting IL-10 and maintaining the state of autoimmune tolerance, which plays a key role in various inflammation diseases therapy [47]. For instance, Nie et al. reported that TNF-α in the synovium of RA patients could inhibit the phosphorylation of FoxP3, restrain the proliferation of Treg and secretion of functional cytokines, leading the occurrence and development of RA [49]. Shi *et al. in situ* injected a nanoparticle drug delivery system loading IL-2, TGF-β, and cyclin dependent kinase inhibitor to induce Treg differentiation and decrease Th17 production in RA model, thus reducing the secretion of TNF-α in the knee, which facilitated to relieve the severity and progression of arthritis [50]. Recently, tolerant DC infusion has been developed to induce Treg and inhibit inflammatory macrophages during inflammatory response. In our previous study, we demonstrated that the adoptive transfer of endotoxin tolerant DC into septic mice could reduce the secretion of inflammatory factors to relieve pathological injuries [12, 51]. However, the immunomodulatory effect of tolerant DC infusion in clinical studies was not optimal, primarily due to the poor cell survival of tolerant DC in vivo.

In recent years, EV mainly refer to exosomes that have emerged as the potential cells-free therapeutics due to their high biostability and bioactive ingredients such as mRNA, miRNA, and cytokines, which have a similar effect to those parental cells and can modulate the biological activities of recipient cells [52]. For instance, Zhang et al. reported that hydrogel loaded DC-derived exosomes could activate Treg and improve cardiac function after myocardial infarction and Zhang et al. also utilized DC-derived exosomes to upregulate Treg cells in the thymuses of rats in experimental autoimmune myasthenia gravis [53, 54]. However, the clinical applications of EV have been limited since they are mostly eliminated by the liver, resulting in a short blood circulation time after systemic administration [23]. Therefore, increasing the accumulation in immune organ (spleen or lymph node) and then improving the selective T-lymphcyte targeting maybe a potential strategy for DC-derived EV to induce Treg, thereby managing inflammatory responses to infections or tissue injury to maintain immune homeostasis.

Unlike exosomes derive from endosome, MV are formed by the direct outward budding of the plasma membrane [24]. The unique features of MV allow them to particularly inherit a significant portion of surface receptors and signalling proteins from their parental cells, thereby endowing MV can act as potent mediators of intercellular communication to influence cellular functions and disease progression [25, 26]. However, it is true that there is relatively limited research on the specific immune regulation abilities of MV and the potential applications of MV-derived biomaterials. Nonetheless, emerging studies are beginning to shed light on these aspects. In this study, inspired by the natural origin of MV, we proposed a means to mimic tolerant DC-derived MV using a simple and effective extrusion method. We proved that this approach could produce a large amount of MVM_chDC_, and would enhance the specific surface protein (such as PD-L1 and azide group) of MVM_chDC_ and bioactive cargos (such as proteins and miR-155-3p), thus improving their homing ability to spleen. The spleen contains a variety of immune cells, for instance, B cells, T-lymphcytes, and monocytes, etc [55]. . To obtain the specific T cells-targeting ability, we conjugated αCD3 antibodies on the surface of MVM_chDC_ through metabolic glycoengineering and bioorthogonal copper-free click chemistry (Fig. 1). Confocal microscope imaging showed the uptake of MVM^αCD3^_chDC_ in T cells was promoted by αCD3 antibodies (Fig. 2). Furthermore, the accumulation and T cells-targeting of MVM^αCD3^_chDC_ in spleen after intravenous injection were confirmed by microscopy and FACS analysis (Fig. 2). These results suggested that MVM^αCD3^_chDC_ with similar structure and homing ability as natural MV could be produced using a straightforward extrusion method. Moreover, the surface modification of αCD3 antibodies significantly enhanced their targeting ability towards T cells within the spleen.

Anti-inflammatory M2 macrophage and Treg are important innate and adaptive immune cells in immunosuppression, respectively [56, 57]. We found that MVM^αCD3^_chDC_ had the ability to promote the induction of CD3^+^CD4^+^FoxP3^+^ Treg and increase the secretion of IL-10 (Fig. 3). These findings suggested that MVM^αCD3^_chDC_ had the potential for the treatment of acute or chronic inflammatory diseases by improving the peripheral immunosuppression. Further studies demonstrated that MVM^αCD3^_chDC_ promoted the phenotype polarization of M0 macrophages towards anti-inflammatory F4/80^+^CD206^+^ M2 macrophages, inhibited the pro-inflammatory F4/80^+^CD86^+^ M1 macrophages, and increased the secretion of anti-inflammatory cytokines in M2 macrophages (Fig. 3). These results indicated that MVM^αCD3^_chDC_ largely inherited the immunosuppressive function of chDC. However, little information is available regarding the mechanism of DC-derived MV for Treg induction. Therefore, we analysed the transcriptomic profiles of MVM_chDC_ and found many significantly expressed miRNAs (e.g., miR-155-3p, miR-125a-3p and miR-125a-5p) that were associated with Treg upregulation (Fig 0.4). Lu et al. observed that miRNA-155 deficiency in Treg results in increased SOCS1 expression accompanied by impaired STAT5 activation and inhibit FoxP3 expression [40, 41]. Especially, both of miR-155-3p and its hairpin (mir-155) were markedly increased in MVM_chDC_ and had a strong effect toward Treg induction. We also showed that miR-155-3p in chDC or MVM_chDC_ was significantly higher than that in imDC or EM_chDC_, respectively, suggesting that MVM prepared by our approach had the ability to carry more bioactive cargos inherited from parental cells compared to EM. In the presence of miR-155-3p mimic and miR-155-3p inhibitor further verified miR-155 could improve FoxP3 expression in Treg by inhibiting SOCS1 pathway. The role of miR-155-3p in MVM_chDC_ and Treg induction may offer an unique perspective for the development of EV-based immnosuppression therapy. Although there has been controversy surrounding miR−155, several studies have demonstrated its pivotal role in autoimmune diseases by promoting pathogenic Th17 responses [58–62]. However, we didn’t observe any alteration in the differentiation of Th17 cells after MVM treatment both in *vivo* and in *vitro* (Figs. S18 and S21), which was consistent with the findings of Lu et al. [41]. They suggested that the repression of SOCS1 by miR−155 is not necessary for the promotion of Th17 differentiation. Based on our data, we concluded that miR−155 could regulate FoxP3 by targeting SOCS1 in Treg cells. However, further research is needed to explore the underlying mechanisms in detail. Last, in both acute inflammation disease model (sepsis) and chronic autoimmune disease model (RA), MVM^aCD3^_chDC_ promoted the increase of Treg in spleen and lymph node, and reduced the concentration of pro-inflammatory cytokines in the serum, thus improving the survival rate of septic mice and ameliorating joint injury RA mice (Figs. 5 and 6).

## Conclusions

In summary, to mimic the natural DC-derived MVs and further enhance the targeting ability to T cells, we developed MVM^αCD3^_chDC_ through the combination of extrusion and surface engineering. Our design enabled MVM^αCD3^_chDC_ to preserve the membrane surface proteins of chDC and their homing ability to spleen, and selectively targeting T-lymphcytes within spleen. Additionally, our studies established that MVM^αCD3^_chDC_ could increase the induction of Treg and promote the polarization of M2 macrophage, leading to the improvement of anti-inflammatory cytokines secretion. Mechanistically, the elevated Treg levels induced by MVM_chDC_ were mediated through the increased miR-155-3p. Upon systemic administration, MVM^αCD3^_chDC_ showed excellent immunosuppressive efficiency in both models of sepsis and RA. Therefore, our work presented a novel cell-free therapeutic system with broad applications for the treatment of inflammatory and autoimmune diseases.

## Methods

### Materials

RPMI-1640, penicillin streptomycin, trypsin, bicinchoninic acid (BCA) protein assay kit and cell counting kit-8 (CCK-8) were purchased from Meilunbio (Dalian, China). Fetal bovine serum (FBS) was purchased from ExCell Bio (Shanghai, China). Lipopolysaccharide and 1,1’-dioctadecyl-3,3,3’,3’-tetramethylindodicarbocyanine,4-chlorobenzenesulfonate salt (DiD) fluorochrome were purchased from Sigma-aldrich. Granulocyte-macrophage colony-stimulating factor (GM-CSF), Interleukin (IL) -2, IL-4, IL-13, and interferon-γ (IFN-γ) were purchased from Peprotech (USA). Dibenzocycolctyne-cyanine5 (DBCO-Cy5), N-azidoacetylmannosamine-tetraacylated (Ac_4_ManNAz) and dibenzylcyclooctyne- NHS ester (DBCO-NHS) were purchased from Macklin (Shanghai, China). Goat Anti-Rat IgG H&L (FITC) and anti-SOCS1 antibody were purchased from Abcam. Type II bovine collagen, Freund’s complete adjuvant and Freund’s incomplete adjuvant were purchased from Chondrex (Washington, USA).

### Animals

C57BL/6 (female, 6–8 weeks) and DBA/1 mice (male, 6–8 weeks) were purchased from Zhejiang Vital river Experimental Animal Technology Co. Ltd. All animals were treated in compliance with the guidelines in the Guidance Suggestions for the Care and Use of Laboratory Animals. This research was approved by the Experimental Animal Ethics Committee of Wenzhou Institute, University of Chinese Academy of Sciences.

### BMDC culture and stimulation

BMDC were isolated from the C57BL/6 mice according to an reported method [31]. Briefly, femur and tibiae were isolated from muscle tissue of mice. The intact bones were then sterilized with 70% ethanol for 5 min and washed with phosphatebuffered saline (PBS). Bone ends were cut and the bone marrow was flushed with PBS. Cellular clusters in the bone marrow suspension were disintegrated and washed with PBS. Monocytes from bone marrow were cultured in RPMI-1640 complete medium including penicillin (100 U/mL), streptomycin (100 U/mL) and 10% inactivated FBS, with GM-CSF (20 ng/mL) and IL-4 (5 ng/mL) to obtain BMDC. A fresh complete medium change was performed on days 4. For immature DC (imDC), cells were collected at day 7 without other treatment. For prime DC (prDC), cells were treated with LPS for 48 h at 0.1 µg/mL at day 5. For challenge DC (chDC), cells were treated with LPS for 48 h at 0.1 µg/mL at day 5, and then treated with LPS for further 24 h at 1 µg/mL. For mature DC (mDC), cells were treated with LPS for 24 h at 1 µg/mL at day 7. Ac_4_ManNAz was added to medium and incubated with chDC for 3 days to obtain chDC^Azido^.

### Preparation and characterization of MVM

DC-derived MVM_imDC_, MVM_chDC_ and MVM^Azido^_chDC_ were collected using the extrusion method [27]. Briefly, imDC, or chDC, or chDC^Azido^ (1 × 10^6^ cells) were collected and resuspended in 1 mL of PBS. The cell suspensions were extruded through 5 μm polycarbonate membrane filters (Nuclepore, Whatman Inc., Clifton, NJ) using a mini-extruder (Avanti Polar Lipids, Birmingham, AL). Cell debris of the final extruded sample (1 mL) were removed by centrifugation at 1,000 ×g for 10 min at 4 ℃. The MVM_imDC_, or MVM_chDC_, or MVM^Azido^_chDC_ were collected at 21,000 ×g for 30 min at 4 ℃. The final sample was stored at -80 ℃. The MVM^αCD3^_chDC_ were obtained by conjugating DBCO-αCD3 antibody onto the surface of MVM^Azido^_chDC_. Briefly, 3 mM of DBCO-NHS in methyl alcohol was added into 0.6 mM of αCD3 antibody (Biolegend, USA) in PBS and swirled for 5 min at room temperature following overnight incubation at 4 °C. 1 µM DBCO-αCD3 antibody was added to the MVM^Azido^_chDC_ suspension to react for 2 h at 4 °C, then unreacted DBCO-αCD3 antibody were removed using centrifugation. For EM preparation, the cell suspensions were extruded sequentially through 5-, 1- and 0.2 μm polycarbonate membrane filters using a miniextruder. Cell debris and microvesicles of the final extruded sample were removed using centrifugation at 10,000 ×g for 10 min at 4 ℃. The EM were purified and concentrated with a 100 kDa centrifugal filter (EMD Millipore, Temecula, CA, USA) at 1,000 ×g for 15 min at 4 ℃ with PBS.

To evaluate the azido positive percentage of chDC^Azido^, chDC were incubated with Ac_4_ManNAz at different concentrations for desired time duration. Then, chDC^Azido^ were labeled by DBCO-Cy5 and examined using flow cytometry. Similarly, to evaluate the azido positive percentage of MVM^Azido^_chDC_, chDC^Azido^ were extruded to obtain MVM^Azido^_chDC_, and then MVM^Azido^_chDC_ were labeled by DBCO-Cy5 and were examined using flow cytometry. The size and ζ-potential of MVM^αCD3^_chDC_ were analyzed by DLS using Nano ZS (Malvern). The morphology of MVM^αCD3^_chDC_ was further observed by transmission electron microscopy (TEM, Thermo, Talos F200S). To confirm the conjugation of αCD3 antibody on the surface of MVM^Azido^_chDC_*via* click chemistry reaction, MVM^αCD3^_chDC_ were stained with goat anti-rat IgG (FITC, Beyotime, Shanghai, China) and then examined using flow cytometry.

The cytotoxicity of MVM in splenic lymphocytes were examined using CCK-8 assay. Murine primary splenic lymphocytes were collected as the previous reporting [63]. Briefly, the spleens from C57BL/6 mice were processed to obtain single-cell suspensions. Then cells were seeded in a 96-well plate at a density of 5 × 10^4^ cells under RPMI-1640 complete medium with IL-2 (10 ng/mL). After 72 h incubation with different MVM, the cells were added fresh medium containing 10% CCK-8 reagent for 2 h. The optical density (OD) at 450 nm was measured with a microplate reader (ELx800, BioTek, USA). The protein concentration of MVM was determined using BCA assay. MVM with 80 µg/mL proteins (MVM prepared from 1 × 10^6^ cells = 80 µg) were utilized in the following in vitro experiments. In in vivo experiments, MVM with 160 µg proteins was intravenously injected in mouse each time.

### Intracellular uptake of MVM in splenic lymphocytes

Splenic lymphocytes were seeded in a 24-well plate at a density of 5 × 10^5^ cells in RPMI-1640 complete medium with IL-2 (10 ng/mL). The cellular uptake of different MVMs were detected using flow cytometry and confocal laser scanning microscope (CLSM). DiD fluorochrome was utilized to label MVM_chDC_ and MVM^αCD3^_chDC_. Labeled MVM were incubated with cells for 8 h. Then, the cells were collected and stained for flow cytometry analysis. For CLSM observation, the cells were fixed in 4% paraformaldehyde for 15 min, and then they were stained using anti-CD3ε antibodies and secondary antibodies. Finally, they were observed using a CLSM (Nikon).

### In vivo biodistribution study

EM_chDC_, MVM_chDC_ or MVM^αCD3^_chDC_ labeled with DiD were injected into mice *via* tail vein injections. The mice were euthanized and the major organs (heart, liver, spleen, lung, kidney and inguinal lymph nodes) were harvested after a predetermined time. The harvested organs were washed with PBS before imaging of fluorescence on the IVIS Lumina XRMS Series III spectrum imaging system (the excitation wavelength of 640 nm and emission wavelength of 670 nm).

### In vitro immune cells responses

Splenic lymphocytes and BMDM were isolated from the C57BL/6 mice. Splenic lymphocytes were seeded in a 24-well plate at a density of 5 × 10^5^ cells and incubated with different MVM, miR-155-3p mimics (100 nM, General bio, Anhui, China) and miR-155-3p inhibitors plus MVM_chDC_ at 37 ºC for 72 h. Lipofectamine 3000 (Invitrogen, USA) was used for transfecting according to the manufacturer’s protocol. Subsequently, the cells were tested using flow cytometry and qPCR. The supernatant was tested using ELISA (Peprotech, USA).

To obtain M1 macrophages, BMDM in day 7 were seeded in a 24-well plate at a density of 1 × 10^5^ cells, and then LPS (100 ng/mL) and IFN-γ (20 ng/mL) were added in medium for further 24 h incubation. To obtain M2 macrophages, IL-4 (10 ng/mL) and IL-13 (10 ng/mL) were added in medium for further 48 h incubation. BMDMs, or M1, or M2 macrophage were incubated with different MVMs at 37 ºC for 48 h. Subsequently, the cells were tested using flow cytometry and qPCR. The supernatant was tested using ELISA (Peprotech, USA).

In flow cytometry measurement, antibodies against CD11b, CD11c, F4/80, Ly-6G, CD86, CD206 and PD-L1 (Biolegend, USA) were utilized for myeloid cell staining. Antibodies against CD3ε, CD4 and FoxP3 utilized for T cell staining (Biolegend, USA). Cytofix/cytoperm soln kit (BD bioscience) and FoxP3 transcription factor staining buffer set kit (Invitrogen) were used for CD206 staining and FoxP3 staining, respectively. All data were collected on CytoFLEX (Beckman) and were analyzed with FlowJo software (Tree Star, Inc.). In qPCR measurement, the cDNA of mRNA were synthesised using PrimeScript RT Reagent Kit (TaKaRa). The expression of mRNA was detected using BlasTaq 2×qPCR MasterMix (Abm, Jiangsu, China). The gene-specific primers were listed in Table S1. The relative expression of mRNA normalised to GAPDH was calculated using the 2^-ΔΔCt^ method.

### RNA extraction and transcriptomic sequencing

Three biological replicates were carried out based on the treat group MVM_chDC_ and the control group MVM_imDC_, respectively. The six samples (two groups by triplets) were then subject to high-throughput transcriptomic sequencing. After total RNA was extracted by Trizol reagent kit (Invitrogen), the RNA molecules in a size range of 18–30 nt were enriched by polyacrylamide gel electrophoresis (PAGE). Then the 3′ adapters were added and the 36–48 nt RNAs were enriched. The 5′ adapters were then ligated to the RNAs as well. The ligation products were reverse transcribed by PCR amplification and the 140–160 bp size PCR products were enriched to generate a cDNA library and sequenced using Illumina HiSeq X Ten System by Gene Denovo Biotechnology Co. (Guangzhou, China). The raw data were processed and analyzed using the nf-core rnaseq (v3.12.0) and smrnaseq (v2.2.1) pipelines and the differentially expressed genes were obtained by edgeR R package (version 3.42.4). In the pipelines, sequenced reads were mapped to the mouse genome (version GRCm39). The genomic annotations were obtained from the GENCODE mouse gene annotation (Release M31) and the miRNA and hairpin annotations were obtained from the miRbase database [64]. We identified the differentially expressed genes and miRNAs with the cut off of log_2_(fold change) > 1 or < -1 and the false discovery rate (FDR) adjusted *P*-value) < 0.1. The volcano plots were generated by Enhanced Volcano R package (v1.18.0). The pathway (KEGG and GO) enrichment analyses were performed by Enrichr tool [65].

The cDNAs of miRNAs were synthesised using First Strand cDNA Synthesis kit (Stem-loop Method). The relative expression of miRNA normalised to U6 controls was calculated using the 2^-ΔΔCt^ method.

### Western blot assay

The activity of SOCS1 was detected by western blot assay. Proteins were extracted using RIPA lysis buffer, and their concentrations were determined *via* the BCA assay. Protein extracts were separated using 12.5% SDS-polyacrylamide gel and then their were transferred to PVDF membranes (Roche). After blocking with 3% BSA solution for 2 h, the membranes were incubated with primary antibodies at 4 ºC overnight. The membranes were washed with tris-buffered saline containing 0.1% Tween-20 and incubated with secondary antibody for 2 h at room temperature. The immunoreactivity was visualized using ECL luminescence reagent (Meilunbio, Dalian, China).

### Sepsis model establishment and treatment

CLP were used for sepsis model establishment. Briefly, after the mice were anesthetized, their abdominal hair was shaved, and 1.0 cm of the skin was cut with a scalpel. The cecum was ligated using a 4 − 0 suture. The cecal contents were gently pushed to the distal end with tweezers, and the cecum was punctured with a 18G needle. Subsequently, a small amount of fecal drop was squeezed from the puncture hole. The cecum was then placed back into the abdomen and the incision was closed. Mice were treated with PBS, MVM_imDC_, MVM_chDC_, and MVM^αCD3^_chDC_ by tail vein injection as described, respectively (Fig. 5A). After 5 days, these mice were euthanized and the major organs, blood, and bilateral inguinal lymph nodes were harvested for further study. Spleen and lymph nodes were collected for flow cytometry analysis. To investigate the cytokine levels in blood, the collected blood samples were centrifuged to obtain serum for ELISA testing. The major organs were fixed in 4% paraformaldehyde and embedded in paraffin. Paraffin-embedded tissue was cut into 5 μm-thick sections then deparaffinized, rehydrated and stained with H&E. For survival rate assay, mice in the administration group were intravenously injected with different MVMs every 24 h for 7 days instead of euthanizing at 24 h time point.

### Collagen-induced arthritis (CIA) model establishment and treatment

The CIA mice model was established by double immunization. Briefly, on day 0, mice were injected intradermally at the base of the tail with 100 µL of type II bovine collagen (2 mg/mL) (Chondrex) emulsified in equal volumes of Freund’s complete adjuvant (Chondrex). After 3 weeks, the mice were given a booster immunisation with 100 µL of type II bovine collagen (2 mg/mL) emulsified in equal volumes of Freund’s incomplete adjuvant. Mice were treated with PBS, MVM_chDC_, and MVM^αCD3^_chDC_ by tail vein injection every three days until day 48. These mice were euthanized at day 49, and then the major organs, blood, joint tissues, and bilateral inguinal lymph nodes were harvested for further study. Spleen and lymph nodes were collected for flow cytometry analysis, serum samples for ELISA and the major organs for H&E staining. The knee joints were fixed in 4% paraformaldehyde, decalcified in 50 nM EDTA and embedded in paraffin. Sections were deparaffinized, rehydrated and stained with H&E, SO-FG or immune-fluorescent stain.

### Statistical analysis

All values were expressed as the mean ± SD. Statistically significant differences were analyzed using Student’s *t*-test between two groups or One-way analysis of variance (ANOVA) among the groups by GraphPad Prism 8.0 software. Log-rank tests were used to compare survival between treatment groups. Differences with *P* < 0.05 were considered statistically significant. Statistical significance was assigned as ns (not significant) *P* > 0.05, *P < 0.05, **P < 0.01, ***P < 0.001, and ****P < 0.0001.

### Electronic supplementary material

Below is the link to the electronic supplementary material.

**Additional file 1**: Fig. S1 to S21 and Table S1

## Data Availability

No datasets were generated or analysed during the current study.

## References

[1] Li Z, Feng Y, Zhang S, Li T, Li H, Wang D (2023). A multifunctional nanoparticle mitigating cytokine storm by scavenging multiple inflammatory mediators of sepsis. ACS Nano.

[2] Ha E, Bang SY, Lim J, Yun JH, Kim JM, Bae JB (2021). Genetic variants shape rheumatoid arthritis-specific transcriptomic features in CD4(+) T cells through differential DNA methylation, explaining a substantial proportion of heritability. Ann Rheum Dis.

[3] Suthen S, Lim CJ, Nguyen P, Dutertre CA, Lai H, Wasser M (2022). Hypoxia-driven immunosuppression by Treg and type-2 conventional dendritic cells in HCC. Hepatology.

[4] Lin L, Wang M, Zeng J, Mao Y, Qin R, Deng J (2023). Sequence variation of candida albicans Sap2 enhances fungal pathogenicity via complement evasion and macrophage M2-like phenotype induction. Adv Sci.

[5] Passeri L, Andolfi G, Bassi V, Russo F, Giacomini G, Laudisa C (2023). Tolerogenic IL-10-engineered dendritic cell-based therapy to restore antigen-specific tolerance in T cell mediated diseases. J Autoimmun.

[6] Ohnmacht C, Pullner A, King SB, Drexler I, Meier S, Brocker T (2009). Constitutive ablation of dendritic cells breaks self-tolerance of CD4 T cells and results in spontaneous fatal autoimmunity. J Exp Med.

[7] Gregori S, Tomasoni D, Pacciani V, Scirpoli M, Battaglia M, Magnani CF (2010). Differentiation of type 1 T regulatory cells (Tr1) by tolerogenic DC-10 requires the IL-10-dependent ilt4/hla-g pathway. Blood.

[8] Chu CC, Ali N, Karagiannis P, Di Meglio P, Skowera A, Napolitano L (2012). Resident CD141 (bdca3) + dendritic cells in human skin produce IL-10 and induce regulatory T cells that suppress skin inflammation. J Exp Med.

[9] Colonna M, Navarro F, Bellon T, Llano M, Garcia P, Samaridis J (1997). A common inhibitory receptor for major histocompatibility complex class I molecules on human lymphoid and myelomonocytic cells. J Exp Med.

[10] Fanger NA, Maliszewski CR, Schooley K, Griffith TS (1999). Human dendritic cells mediate cellular apoptosis via tumor necrosis factor-related apoptosis-inducing ligand (trail). J Exp Med.

[11] Min Z, Zeng Y, Zhu T, Cui B, Mao R, Jin M (2021). Lipopolysaccharide-activated bone marrow-derived dendritic cells suppress allergic airway inflammation by ameliorating the immune microenvironment. Front Immunol.

[12] Chen Y, Hou C, Yang N, Yang Y, Chen Y, Kong D (2022). Regulatory effect of jak2/stat3 on the immune function of endotoxin-tolerant dendritic cells and its involvement in acute liver failure. J Clin Transl Hepatol.

[13] Giannoukakis N, Phillips B, Finegold D, Harnaha J, Trucco M (2011). Phase I (safety) study of autologous tolerogenic dendritic cells in type 1 diabetic patients. Diabetes Care.

[14] Benham H, Nel HJ, Law SC, Mehdi AM, Street S, Ramnoruth N (2015). Citrullinated peptide dendritic cell immunotherapy in HLA risk genotype-positive rheumatoid arthritis patients. Sci Transl Med.

[15] Bell GM, Anderson AE, Diboll J, Reece R, Eltherington O, Harry RA (2017). Autologous tolerogenic dendritic cells for rheumatoid and inflammatory arthritis. Ann Rheum Dis.

[16] Zubizarreta I, Florez-Grau G, Vila G, Cabezon R, Espana C, Andorra M (2019). Immune tolerance in multiple sclerosis and neuromyelitis optica with peptide-loaded tolerogenic dendritic cells in a phase 1b trial. Proc Natl Acad Sci USA.

[17] Blazar BR, MacDonald K, Hill GR (2018). Immune regulatory cell infusion for graft-versus-host disease prevention and therapy. Blood.

[18] Xu R, Greening DW, Zhu HJ, Takahashi N, Simpson RJ (2016). Extracellular vesicle isolation and characterization: toward clinical application. J Clin Invest.

[19] van Niel G, D’Angelo G, Raposo G (2018). Shedding light on the cell biology of extracellular vesicles. Nat Rev Mol Cell Biol.

[20] Tang TT, Wang B, Lv LL, Liu BC (2020). Extracellular vesicle-based nanotherapeutics: emerging frontiers in anti-inflammatory therapy. Theranostics.

[21] Jang SC, Kim OY, Yoon CM, Choi DS, Roh TY, Park J (2013). Bioinspired exosome-mimetic nanovesicles for targeted delivery of chemotherapeutics to malignant tumors. ACS Nano.

[22] Lee JR, Park BW, Kim J, Choo YW, Kim HY, Yoon JK (2020). Nanovesicles derived from iron oxide nanoparticles-incorporated mesenchymal stem cells for cardiac repair. Sci Adv.

[23] Zhang G, Huang X, Xiu H, Sun Y, Chen J, Cheng G (2020). Extracellular vesicles: natural liver-accumulating drug delivery vehicles for the treatment of liver diseases. J Extracell Vesicles.

[24] Wang J, Zhuang X, Greene KS, Si H, Antonyak MA, Druso JE (2021). Cdc42 functions as a regulatory node for tumour-derived microvesicle biogenesis. J Extracell Vesicles.

[25] Antonyak MA, Li B, Boroughs LK, Johnson JL, Druso JE, Bryant KL (2011). Cancer cell-derived microvesicles induce transformation by transferring tissue transglutaminase and fibronectin to recipient cells. Proc Natl Acad Sci USA.

[26] Feng Q, Zhang C, Lum D, Druso JE, Blank B, Wilson KF (2017). A class of extracellular vesicles from breast cancer cells activates VEGF receptors and tumour angiogenesis. Nat Commun.

[27] Fan J, Lee CS, Kim S, Chen C, Aghaloo T, Lee M (2020). Generation of small RNA-modulated exosome mimetics for bone regeneration. ACS Nano.

[28] Xu F, Fei Z, Dai H, Xu J, Fan Q, Shen S (2022). Mesenchymal stem cell-derived extracellular vesicles with high PD-L1 expression for autoimmune diseases treatment. Adv Mater.

[29] Tsai HI, Wu Y, Liu X, Xu Z, Liu L, Wang C (2022). Engineered small extracellular vesicles as a fgl1/PD-L1 dual-targeting delivery system for alleviating immune rejection. Adv Sci.

[30] Riazifar M, Mohammadi MR, Pone EJ, Yeri A, Lasser C, Segaliny AI (2019). Stem cell-derived exosomes as nanotherapeutics for autoimmune and neurodegenerative disorders. ACS Nano.

[31] Efimova I, Catanzaro E, Van der Meeren L, Turubanova VD, Hammad H, Mishchenko TA et al. Vaccination with early ferroptotic cancer cells induces efficient antitumor immunity. J Immunother Cancer. 2020;8(2). 10.1136/jitc-2020-001369 PMC7668384 33188036

[32] Manni G, Mondanelli G, Scalisi G, Pallotta MT, Nardi D, Padiglioni E (2020). Pharmacologic induction of endotoxin tolerance in dendritic cells by L-kynurenine. Front Immunol.

[33] Fan Z, Zhu P, Zhu Y, Wu K, Li CY, Cheng H (2020). Engineering long-circulating nanomaterial delivery systems. Curr Opin Biotechnol.

[34] Xiao P, Wang J, Zhao Z, Liu X, Sun X, Wang D (2021). Engineering nanoscale artificial antigen-presenting cells by metabolic dendritic cell labeling to potentiate cancer immunotherapy. Nano Lett.

[35] Ruan S, Erwin N, He M (2022). Light-induced high-efficient cellular production of immune functional extracellular vesicles. J Extracell Vesicles.

[36] Zhou L, Park JJ, Zheng Q, Dong Z, Mi Q (2011). MicroRNAs are key regulators controlling iNKT and regulatory T-cell development and function. Cell Mol Immunol.

[37] Maul J, Alterauge D, Baumjohann D (2019). MicroRNA-mediated regulation of T follicular helper and T follicular regulatory cell identity. Immunol Rev.

[38] Pan W, Zhu S, Dai D, Liu Z, Li D, Li B (2015). MiR-125a targets effector programs to stabilize Treg-mediated immune homeostasis. Nat Commun.

[39] Zhang J, Chen C, Fu H, Yu J, Sun Y, Huang H (2020). MicroRNA-125a-loaded polymeric nanoparticles alleviate systemic lupus erythematosus by restoring effector/regulatory T cells balance. ACS Nano.

[40] Lu LF, Thai TH, Calado DP, Chaudhry A, Kubo M, Tanaka K (2009). Foxp3-dependent microRNA155 confers competitive fitness to regulatory T cells by targeting SOCS1 protein. Immunity.

[41] Lu LF, Gasteiger G, Yu IS, Chaudhry A, Hsin JP, Lu Y (2015). A single miRNA-mRNA interaction affects the immune response in a context- and cell-type-specific manner. Immunity.

[42] Wang S, Gao Y (2021). Pancreatic cancer cell-derived microRNA-155-5p-containing extracellular vesicles promote immune evasion by triggering EHF-dependent activation of AKT/NF-kappab signaling pathway. Int Immunopharmacol.

[43] Mycko MP, Cichalewska M, Cwiklinska H, Selmaj KW (2015). MiR-155-3p drives the development of autoimmune demyelination by regulation of heat shock protein 40. J Neurosci.

[44] Escobar TM, Kanellopoulou C, Kugler DG, Kilaru G, Nguyen CK, Nagarajan V (2014). MiR-155 activates cytokine gene expression in Th17 cells by regulating the DNA-binding protein Jarid2 to relieve polycomb-mediated repression. Immunity.

[45] Qian W, Cao Y (2022). An overview of the effects and mechanisms of m6 a methylation on innate immune cells in sepsis. Front Immunol.

[46] Wu KK, Kuo CC, Yet SF, Lee CM, Liou JY (2020). 5-methoxytryptophan: an arsenal against vascular injury and inflammation. J Biomed Sci.

[47] Jin S, Chen H, Li Y, Zhong H, Sun W, Wang J (2018). Maresin 1 improves the Treg/Th17 imbalance in rheumatoid arthritis through miR-21. Ann Rheum Dis.

[48] Ren S, Liu H, Wang X, Bi J, Lu S, Zhu C (2021). Acupoint nanocomposite hydrogel for simulation of acupuncture and targeted delivery of triptolide against rheumatoid arthritis. J Nanobiotechnol.

[49] Nie H, Zheng Y, Li R, Guo TB, He D, Fang L (2013). Phosphorylation of Foxp3 controls regulatory T cell function and is inhibited by TNF-alpha in rheumatoid arthritis. Nat Med.

[50] Wang L, Wang Y, Liu C, He J, He X, Zhang X (2023). Treg-targeted efficient-inducible platform for collagen-induced arthritis treatment. Mater Today Bio.

[51] Zhang SN, Yang NB, Ni SL, Dong JZ, Shi CW, Li SS (2016). Splenic Cd11c(low)CD45RB(high) dendritic cells derived from endotoxin-tolerant mice attenuate experimental acute liver failure. Sci Rep.

[52] Lin J, Lv J, Yu S, Chen Y, Wang H, Chen J (2022). Transcript engineered extracellular vesicles alleviate alloreactive dynamics in renal transplantation. Adv Sci.

[53] Zhang Y, Cai Z, Shen Y, Lu Q, Gao W, Zhong X (2021). Hydrogel-load exosomes derived from dendritic cells improve cardiac function via Treg cells and the polarization of macrophages following myocardial infarction. J Nanobiotechnol.

[54] Zhang P, Liu RT, Du T, Yang CL, Liu YD, Ge MR (2019). Exosomes derived from statin-modified bone marrow dendritic cells increase thymus-derived natural regulatory T cells in experimental autoimmune myasthenia gravis. J Neuroinflammation.

[55] Huang M, Liu X, Ye H, Zhao X, Zhao J, Liu Y (2020). Metabolic defects in splenic B cell compartments from patients with liver cirrhosis. Cell Death Dis.

[56] Li Y, Gao S, Shi S, Xiao D, Peng S, Gao Y (2021). Tetrahedral framework nucleic acid-based delivery of resveratrol alleviates insulin resistance: from innate to adaptive immunity. Nanomicro Lett.

[57] Wei F, Su Y, Quan Y, Li X, Zou Q, Zhang L (2023). Anticoagulants enhance molecular and cellular immunotherapy of cancer by improving tumor microcirculation structure and function and redistributing tumor infiltrates. Clin Cancer Res.

[58] Xin Q, Li J, Dang J, Bian X, Shan S, Yuan J (2015). MiR-155 deficiency ameliorates autoimmune inflammation of systemic lupus erythematosus by targeting S1pr1 in Faslpr/lpr mice. J Immunol.

[59] Bluml S, Bonelli M, Niederreiter B, Puchner A, Mayr G, Hayer S (2011). Essential role of microRNA-155 in the pathogenesis of autoimmune arthritis in mice. Arthritis Rheum.

[60] O’Connell RM, Kahn D, Gibson WS, Round JL, Scholz RL, Chaudhuri AA (2010). MicroRNA-155 promotes autoimmune inflammation by enhancing inflammatory T cell development. Immunity.

[61] Liu Y, Wan X, Yuan Y, Huang J, Jiang Y, Zhao K (2021). Opposite effects of miR-155 in the initial and later stages of lipopolysaccharide-induced inflammatory response. J Zhejiang Univ Sci B.

[62] Yang L, Zhang C, Bai X, Xiao C, Dang E, Wang G (2020). Hsa_circ_0003738 inhibits the suppressive function of Tregs by targeting miR-562/IL-17a and miR-490-5p/IFN-gamma signaling pathway. Mol Ther Nucleic Acids.

[63] Skwarczynski M, Zhao G, Boer JC, Ozberk V, Azuar A, Cruz JG (2020). Poly(amino acids) as a potent self-adjuvanting delivery system for peptide-based nanovaccines. Sci Adv.

[64] Kozomara A, Birgaoanu M, Griffiths-Jones S (2019). Mirbase: from microRNA sequences to function. Nucleic Acids Res.

[65] Chen EY, Tan CM, Kou Y, Duan Q, Wang Z, Meirelles GV (2013). Enrichr: interactive and collaborative HTML5 gene list enrichment analysis tool. BMC Bioinformatics.

