# Supplementary material for: Immunosuppressive microvesicles-mimetic derived from tolerant dendritic cells to target T-lymphocytes for inflammation diseases therapy
Source: J Nanobiotechnology. 2024 Apr 24;22:201. doi: 10.1186/s12951-024-02470-z (PMC11040880; doi:10.1186/s12951-024-02470-z)
Supplement: Supplementary file 1 — Additional file 1: Fig. S1 to S21 and Table S1 [file 12951_2024_2470_MOESM1_ESM.docx]

Supplementary Information for

Immunosuppressive microvesicles-mimetic derived from tolerant dendritic cells to target T-lymphocytes for inflammation diseases therapy

Minghao Lin^1,2,4†^, Siyun Lei^1,2,3†^, Yingqian Chai^1,2,3^, Jianghua Xu^1,2,3^, Youchao Wang^5^, Chenghu Wu^1,2,3^, Hongyi Jiang^2,3^, Shanshan Yuan^1,2,3^, Jilong Wang^1,2,3^, Jie Lyu^1,2*^, Mingqin Lu^1,2*^, Junjie Deng^1,2,3*^

^1^Joint Centre of Translational Medicine, The First Affiliated Hospital of Wenzhou Medical University, Wenzhou, Zhejiang 325000, China.

^2^Joint Centre of Translational Medicine, Wenzhou Institute, University of Chinese Academy of Sciences, Wenzhou, Zhejiang 325000, China.

^3^Zhejiang Engineering Research Center for Tissue Repair Materials, Wenzhou Institute, University of Chinese Academy of Sciences, Wenzhou, Zhejiang 325000, China.

^4^Wenzhou Traditional Chinese Medicine Hospital, Wenzhou 325000, China.

^5^Chimie ParisTech, PSL University, CNRS, Institute of Chemistry for Life and Health Sciences, Laboratory for Inorganic Chemical Biology, Paris 75005, France.


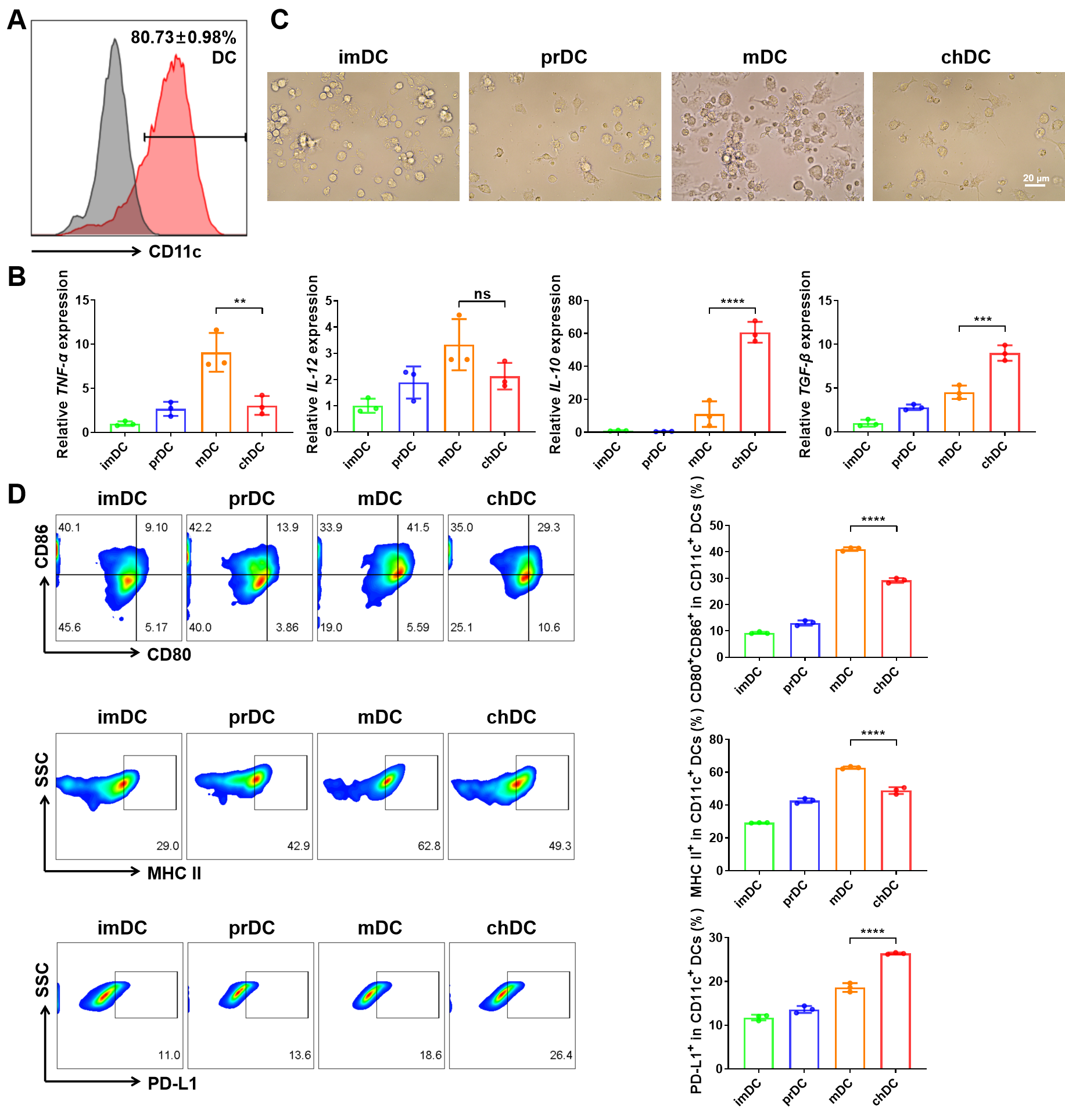


**Fig. S1.** Characterization of murine BMDC. **A** Frequency of dendritic cells in cells from bone marrow after 7 days culture. **B** The expression of TNF-α and IL-10 mRNA was determined by qPCR.  **C** Morphology of BMDC after different stimulation. Scale bar = 20 μm. **D** Percentage of the CD11c^+^CD80^+^CD86^+^ DC, CD11c^+^MHC II^+^ DC and CD11c^+^PD-L1^+^ DC in CD11c^+^ cells using flow cytometry analysis. Data were presented as mean ± SD. Statistical analyses were performed by One-way ANOVA with Tukey method. N = 3 per group. ns *P* > 0.05, **P* < 0.05, ***P* < 0.01, ****P* < 0.001, and *****P* < 0.0001.


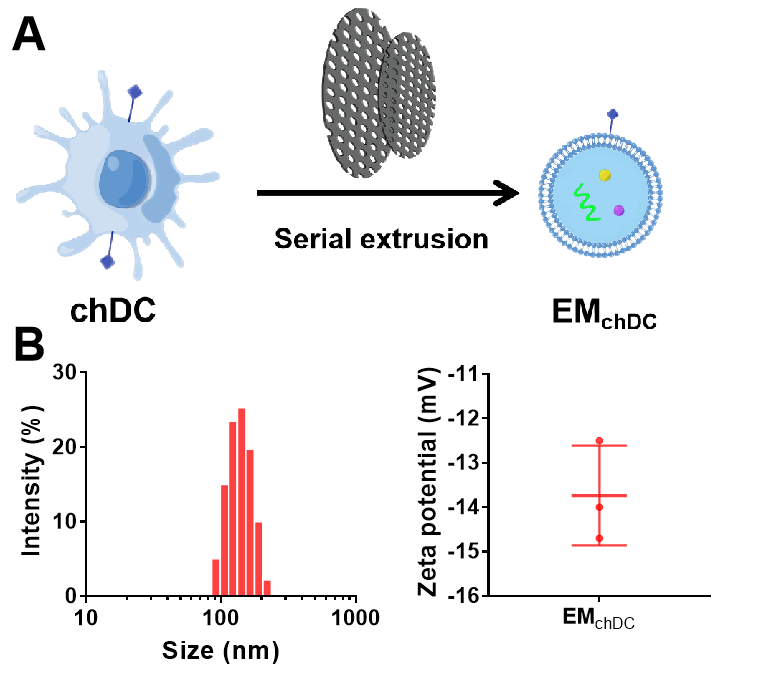


**Fig. S2. A** Schematic of the construction of EM_chDC._ **B** Hydrodynamic diameter and ζ-potential images of EM_chDC_. Data were presented as mean ± SD. N = 3 per group.


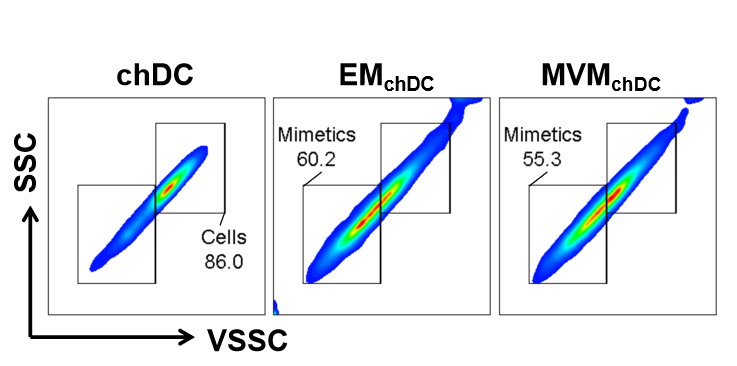


**Fig. S3.** The gate strategy of cells gate on chDC and mimetics gate on EM_chDC_ and MVM_chDC_.


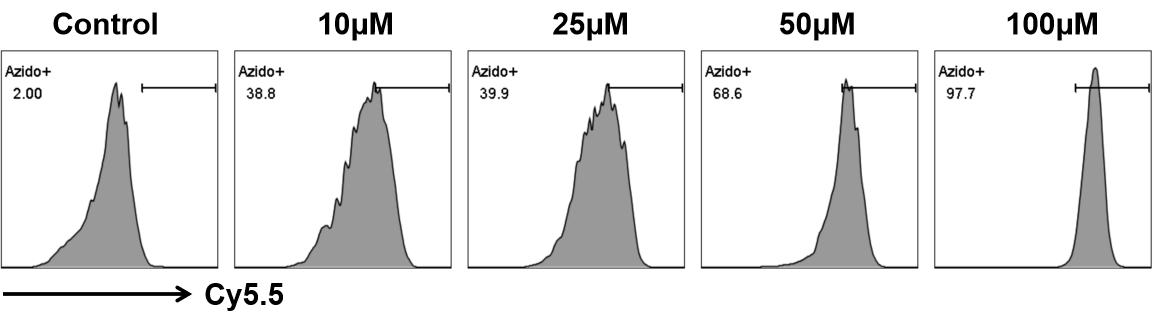


**Fig. S4.** The flow image of azido-labled chDC.


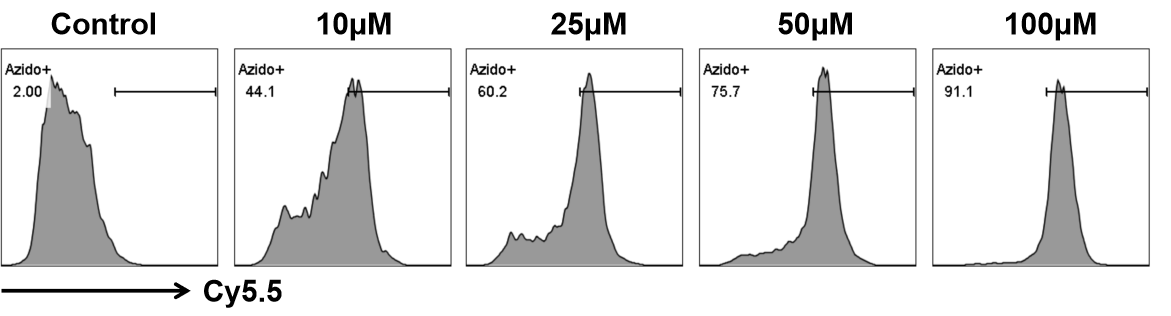


**Fig. S5.** The flow image of azido-labled MVM_chDC_.


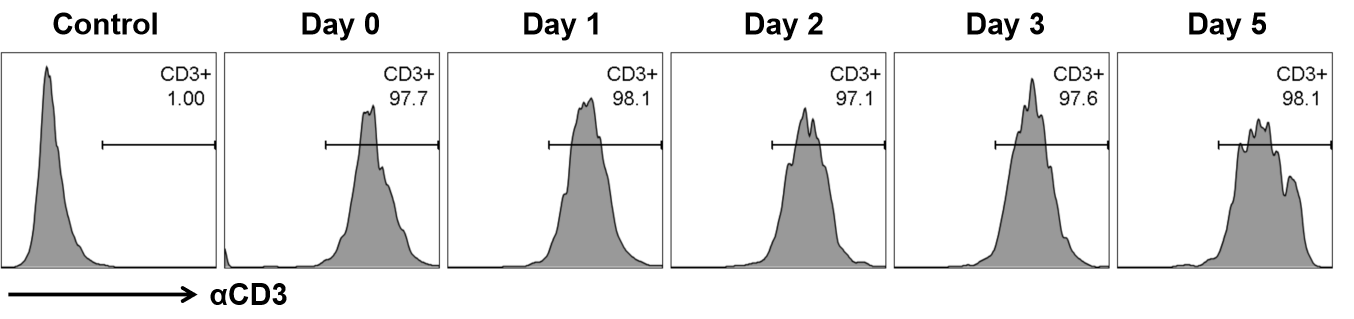


**Fig. S6.** The flow image of αCD3-positive MVM_chDC_.


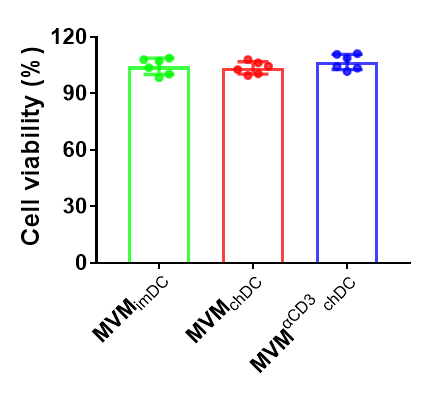


**Fig. S7.** Cytotoxicity viability of splenic lymphocytes after MVM_imDC_, MVM_chDC_ and MVM^αCD3^_chDC_ treatments. Data were presented as mean ± SD. N = 6 per group.


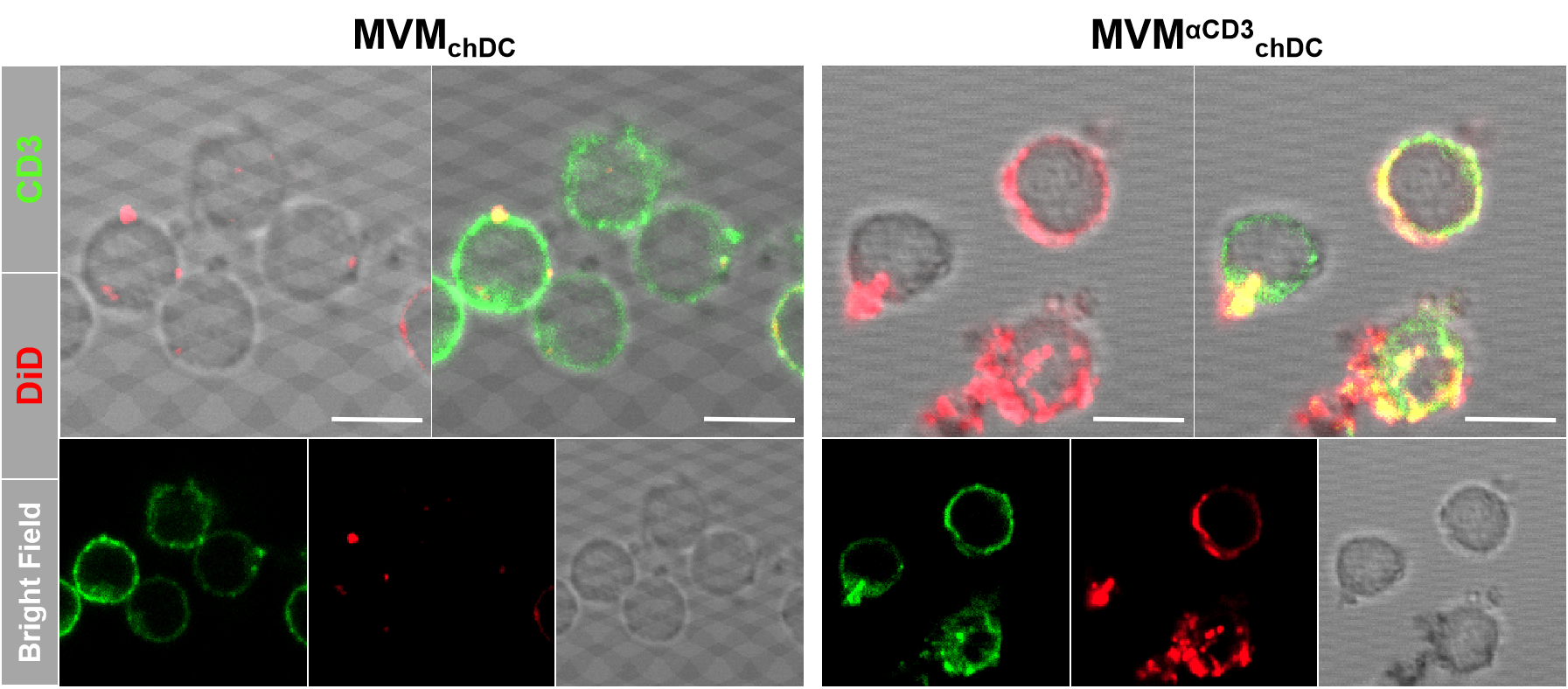


**Fig. S8.** Intracellular uptake of MVM_chDC_ and MVM^αCD3^_chDC_ in T cells after 8 h incubation. MVM_chDC_ and MVM^αCD3^_chDC_ were labeled using DiD (red). T cell membrane was stained with FITC secondary antibody (green). Scale bar = 5 μm.


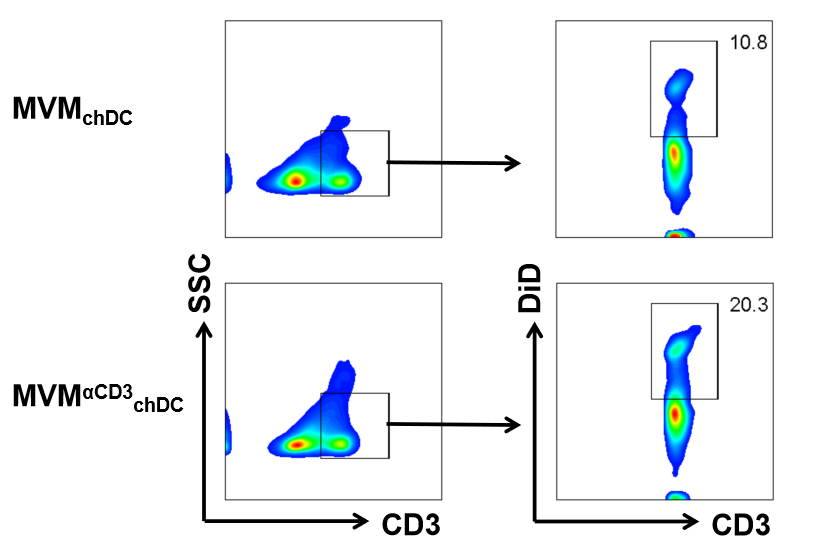


**Fig. S9.** Percentage of the DiD-positive cells in CD3^+^ T cells using flow cytometry analysis.


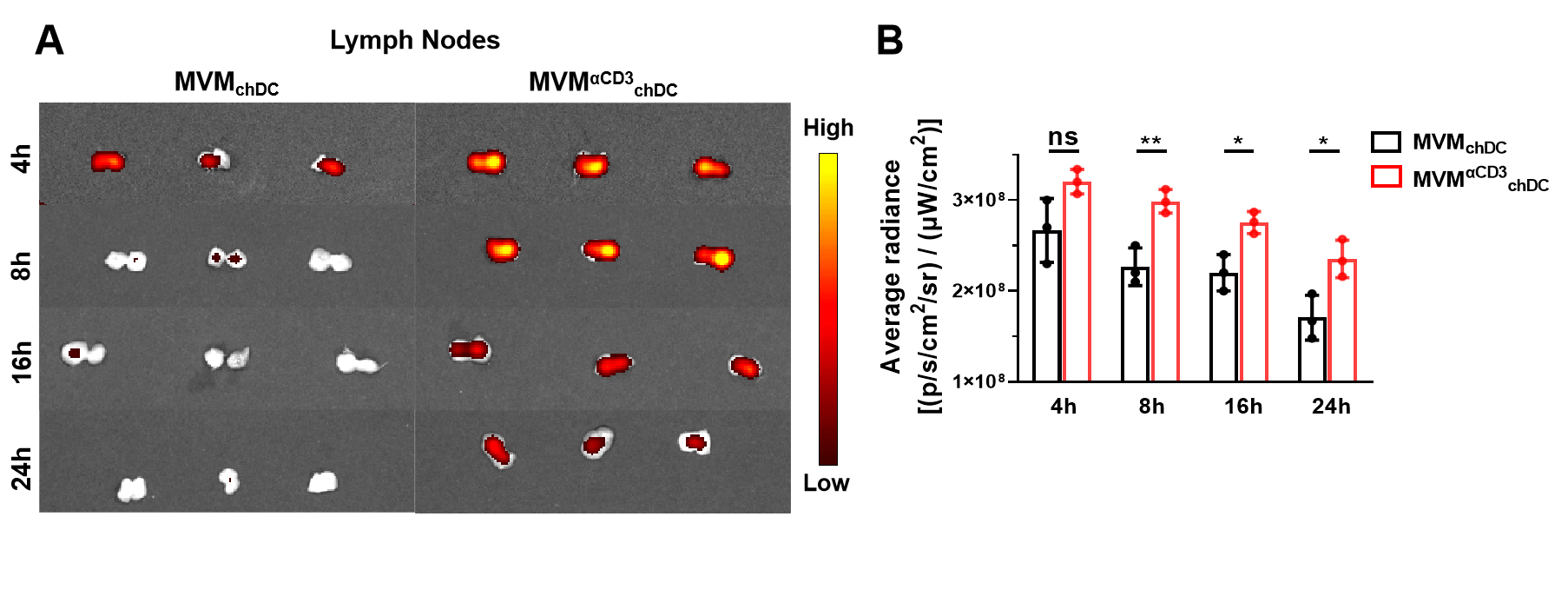


**Fig. S10. A** Lymph node distribution of MVM_chDC_ and MVM^αCD3^_chDC_. Mice were intravenously injected with MVM_chDC_ and MVM^αCD3^_chDC_ at different time points. MVM_chDC_ and MVM^αCD3^_chDC_ were labeled using DiD fluorescence. **B** Quantitative analysis of MVM_chDC_ and MVM^αCD3^_chDC_ in lymph nodes at different time points. Data were presented as mean ± SD. Statistical analyses were performed by Student’s *t*-test. N = 3 per group. ns *P* > 0.05, **P* < 0.05, and ***P* < 0.01.


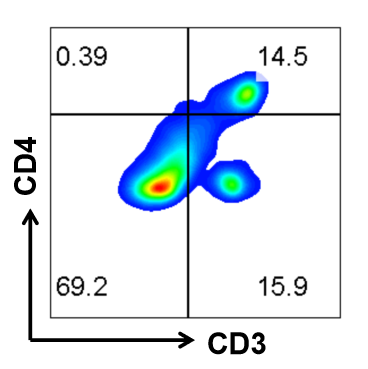


**Fig. S11.** The gate strategy of CD3^+^CD4^+^ T cells from splenic lymphocytes.


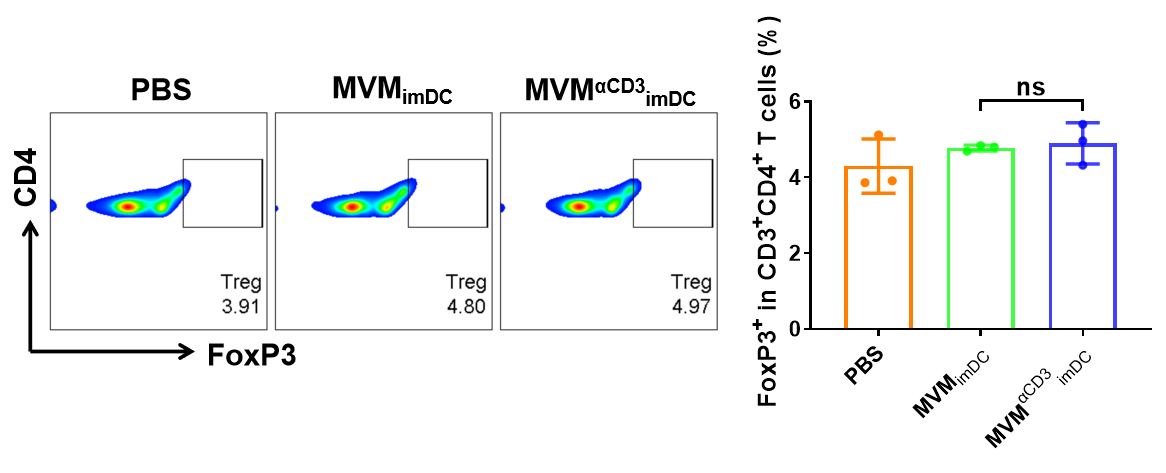


**Fig. S12.** Percentage of the CD3^+^CD4^+^FoxP3^+^ Treg in CD3^+^CD4^+^ T cells using flow cytometry analysis. Data were presented as mean ± SD. Statistical analyses were performed by One-way ANOVA with Tukey method. N = 3 per group. ns *P* > 0.05.


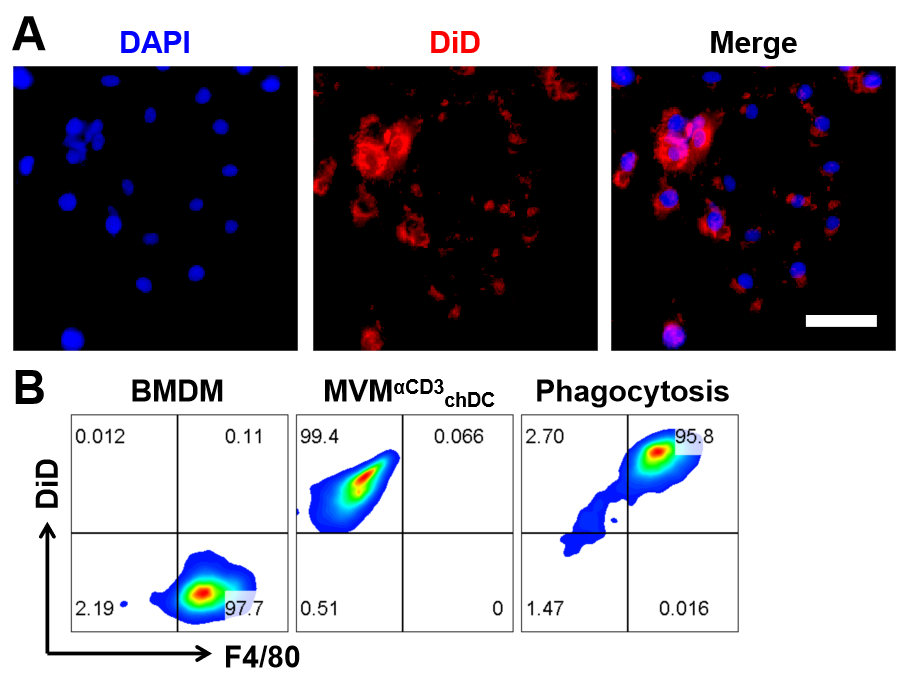


**Fig. S13. A** Intracellular uptake of MVM^αCD3^_chDC_ in BMDM after 2 h incubation. MVM^αCD3^_chDC_ were labeled using DiD (red) and the nucleus were stained with DAPI (blue). Scale bar = 20 μm. **B** The corresponding intracellular fluorescence signals were determined by flow cytometry analysis.


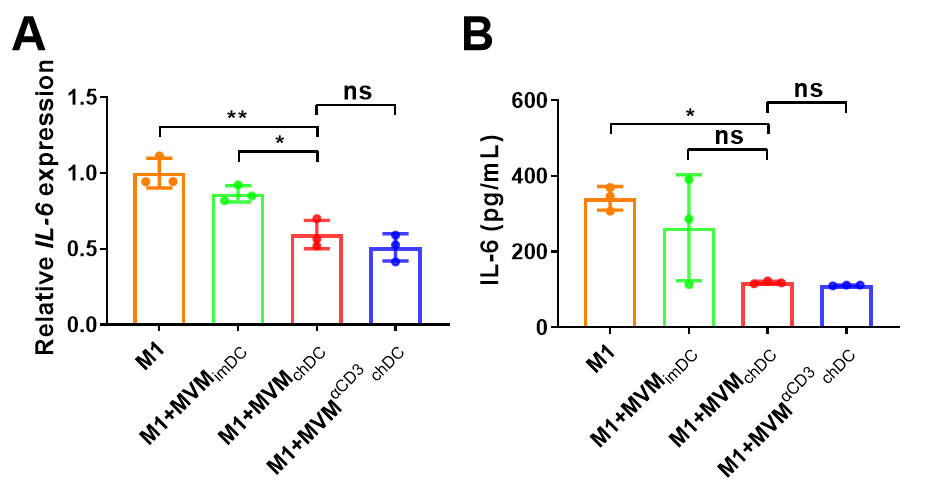


**Fig. S14.** **A** Relative mRNA expression of IL-6 of M1 macrophages after different treatments. **B** The concentration of TNF-α in supernatant of M1 macrophages after different treatments. Data were presented as mean ± SD. Statistical analyses were performed by One-way ANOVA with Tukey method. N = 3 per group. ns *P* > 0.05, **P* < 0.05, and ***P* < 0.01.


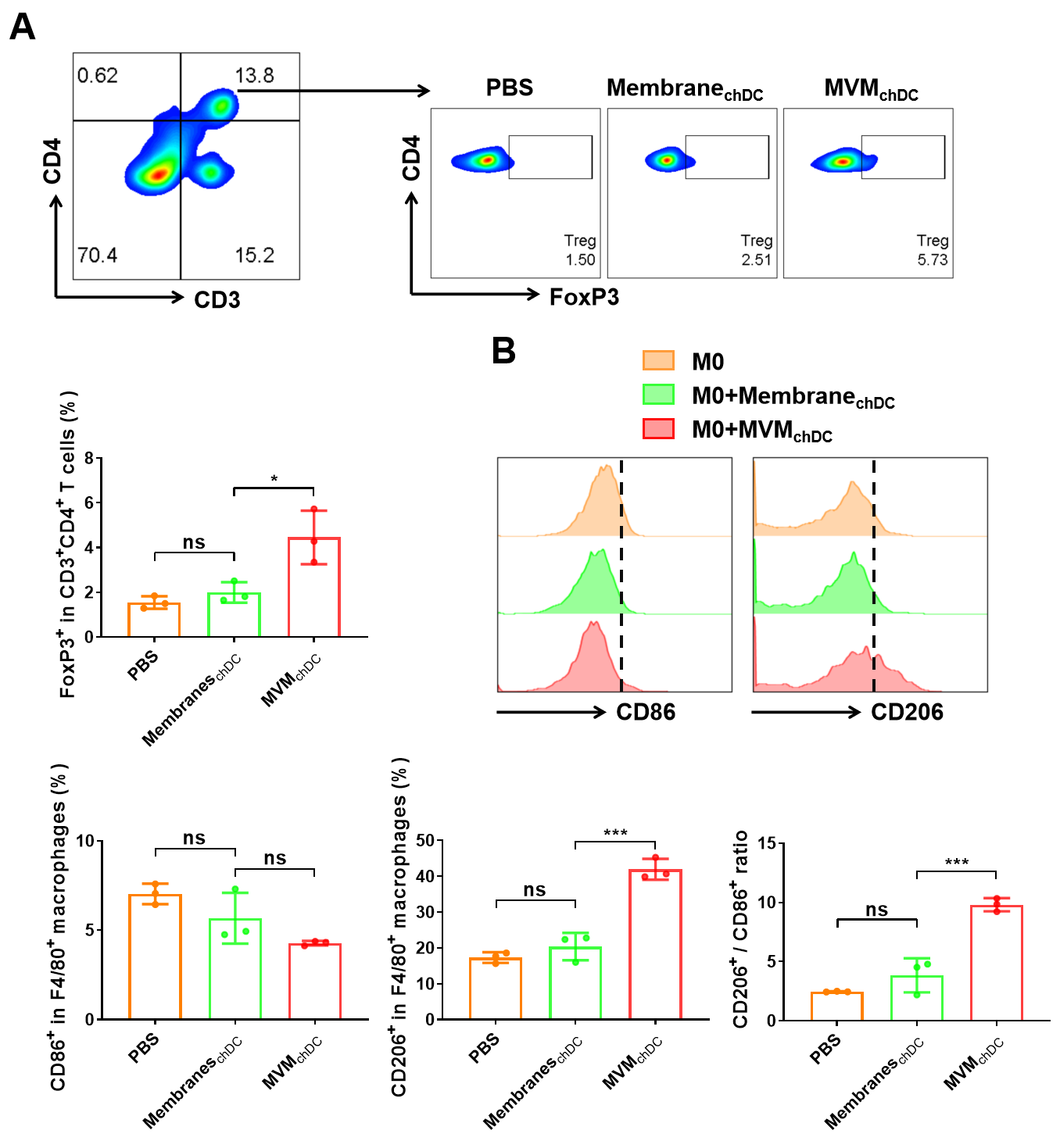


**Fig. S15.** **A** Percentage of the CD3^+^CD4^+^FoxP3^+^ Treg in CD3^+^CD4^+^ T cells using flow cytometry analysis. **B** Percentage of F4/80^+^CD86^+^ M1 and F4/80^+^CD206^+^ M2 in F4/80^+^ macrophages and the corresponding M2/M1 ratio using flow cytometry analysis. Data were presented as mean ± SD. Statistical analyses were performed by One-way ANOVA with Tukey method. N = 3 per group. ns *P* > 0.05, **P* < 0.05, ***P* < 0.01, and ****P* < 0.001.


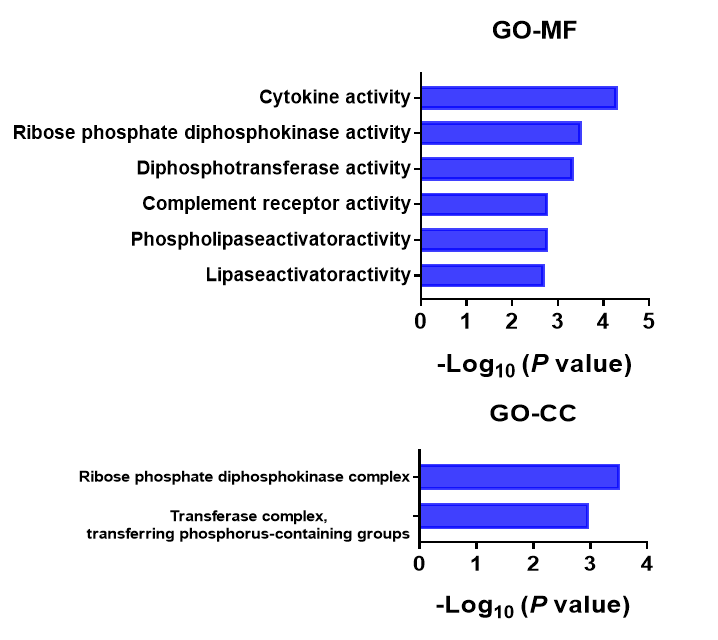


**Fig. S16.** Enriched terms of molecular function and cellular component of up-regulated genes by GO analysis.

**Fig. S17.** Relative expression of miR-155-3p in EM_chDC_ and MVM_chDC_. Data were presented as mean ± SD. Statistical analyses were performed by Student’s *t*-test. N = 3 per group. ns *P* > 0.05, and **P* < 0.05.


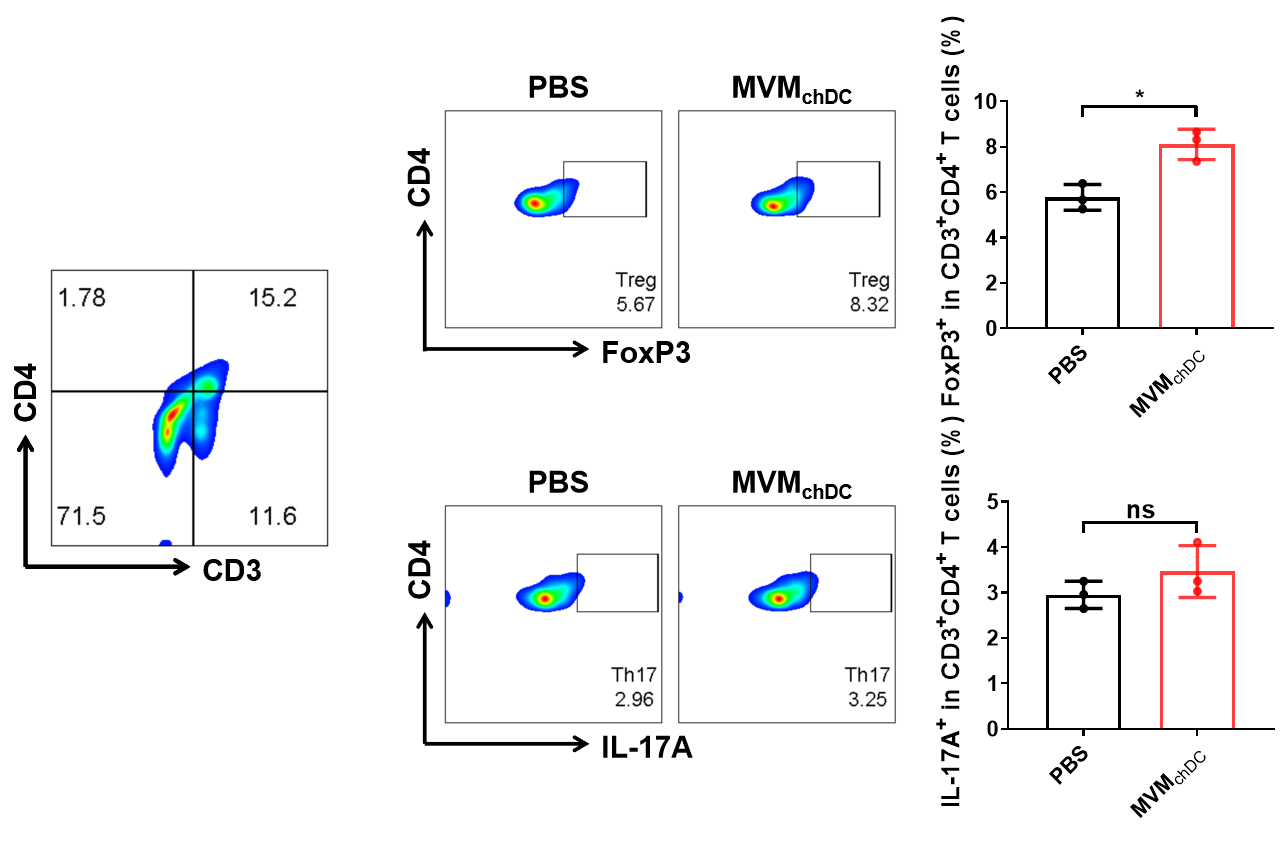


**Fig. S18.** Percentage of CD3^+^CD4^+^FoxP3^+^ Treg and CD3^+^CD4^+^IL-17^+^ Th17 in CD3^+^CD4^+^ T cells using flow cytometry analysis. Data were presented as mean ± SD. Statistical analyses were performed by Student’s *t*-test. N = 3 per group. ns *P* > 0.05, and **P* < 0.05.


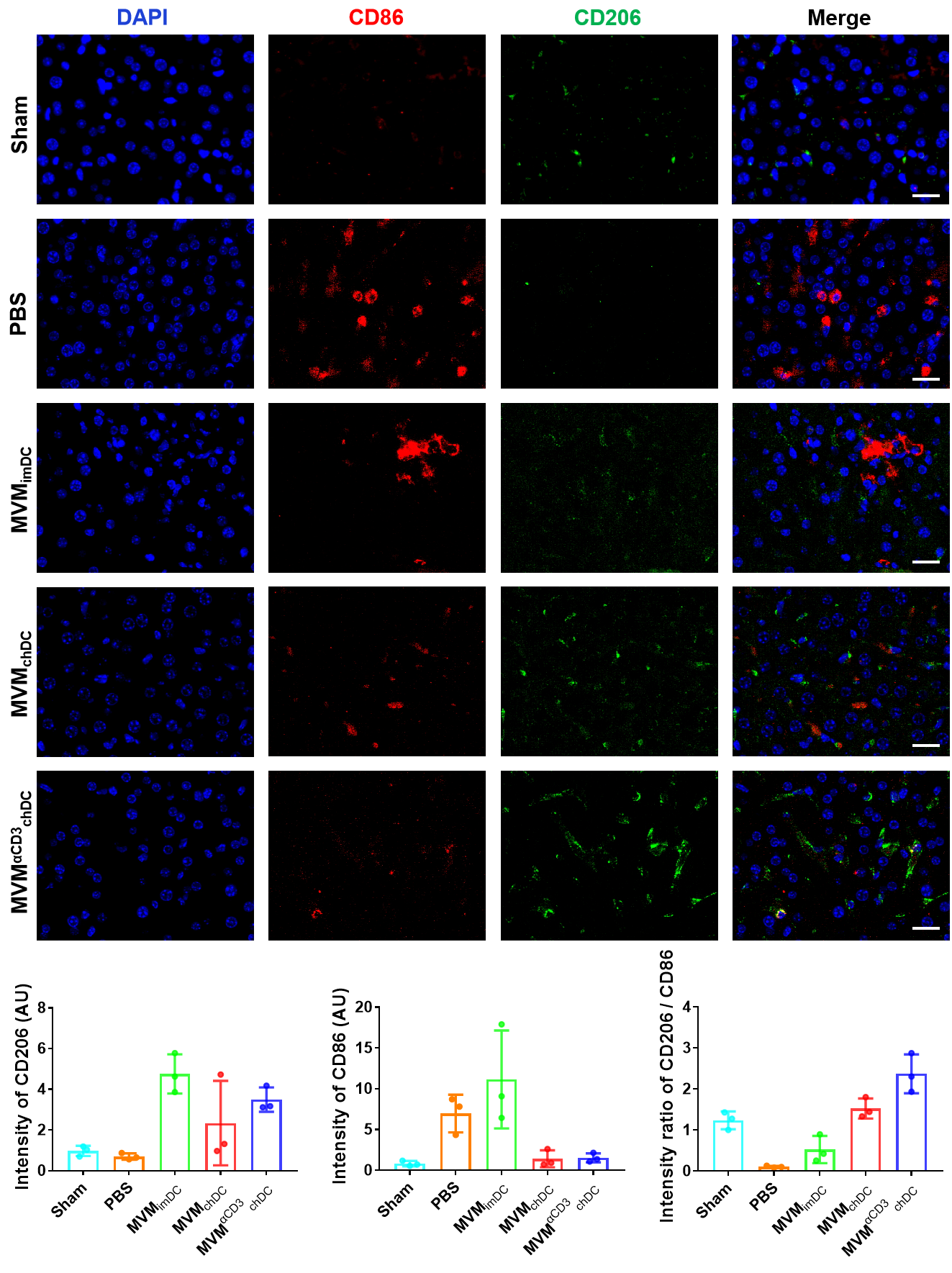


**Fig. S19.** Photomicrographs show the labelling of CD86 (red), CD206 (green) and DAPI (blue). Merged images show the colocalization of DAPI, CD86 and CD206. Scale bar = 20 μm. Data were presented as mean ± SD. N = 3 per group.


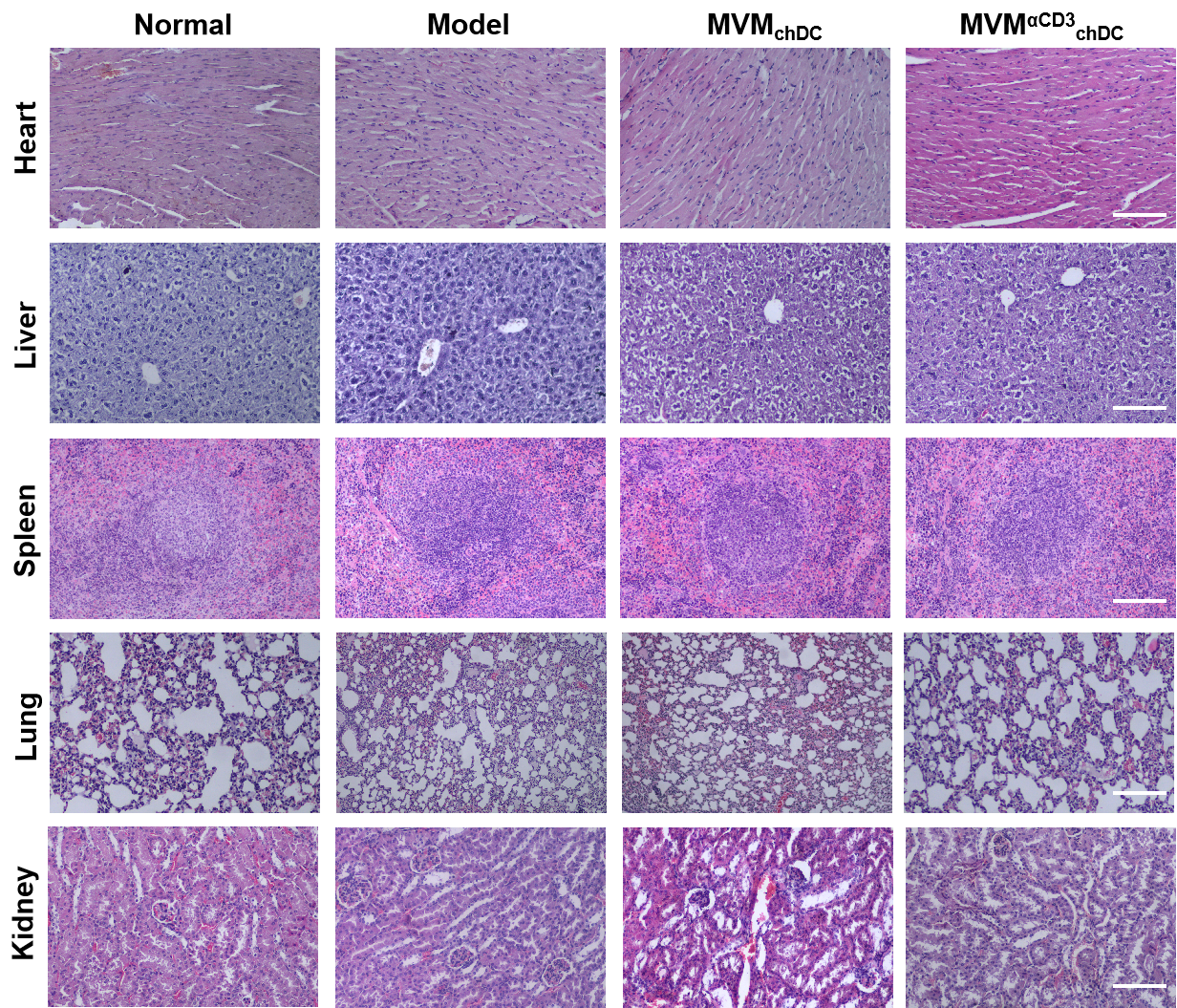


**Fig. S20.** H&E staining of heart, liver, spleen, lung, and kidney tissues after different treatments. Scale bar = 100 μm.


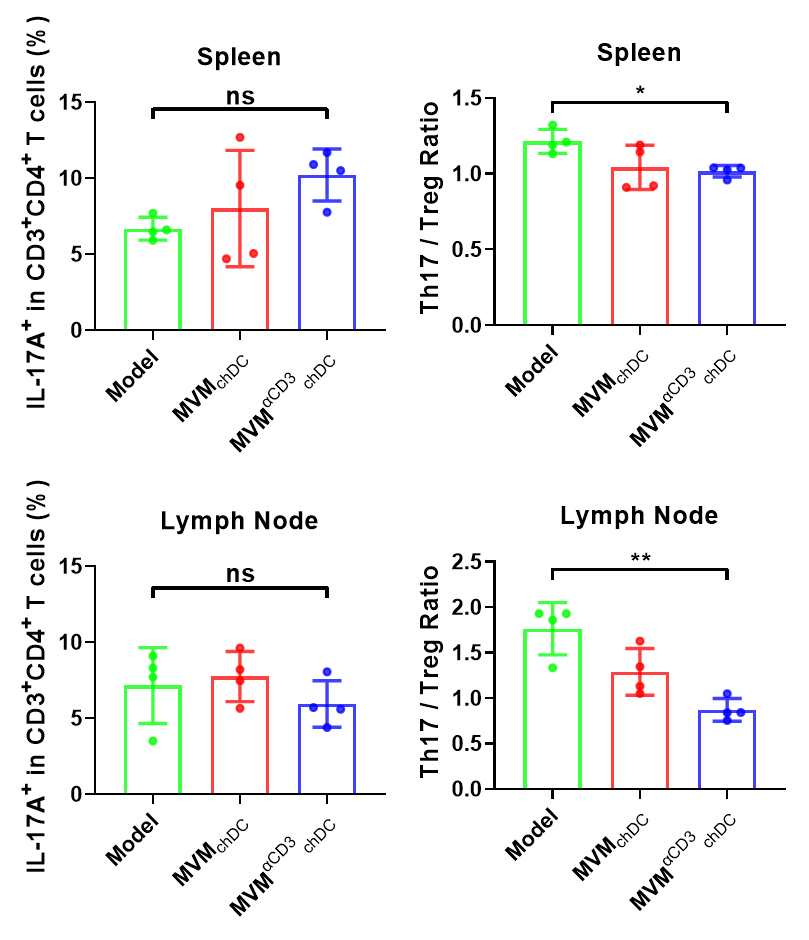


**Fig. S21.** Percentage of CD3^+^CD4^+^IL-17A^+^ Th17 in CD3^+^CD4^+^ T cells within spleen and lymph nodes and the corresponding Th17/Treg ratio using flow cytometry analysis. Data were presented as mean ± SD. Statistical analyses were performed by One-way ANOVA with Tukey method. N = 4 per group. ns *P* > 0.05, **P* < 0.05, and ***P* < 0.01.

**Table S1.** Primer sequence

| **Genes** | **Primer sequence (5’-3’)** |
| --- | --- |
| miR-155-3p | Sequence:CUCCUACCUGUUAGCAUUAAC |
|  | Stem-ring: CTCAACTGGTGTCGTGGAGTCGGCAAT  TCAGTTGAGGTTAATGC |
|  | Forward(F): CTCCTACCTGTTAGCATTAAC |
|  | Reverse(R): TGGTGTCGTGGAGTCG |
| Universal U6 | F: CTCGCTTCGGCAGCACA |
|  | R: AACGCTTCACGAATTTGCGT |
| FoxP3 | F: CACAGAGGGGCAGGCAACAA |
|  | R: GTCACCCCAACACAGCGACA |
| IL-10 | F: GCCAGTACAGCCGGGAAGAC |
|  | R: TCTCTGCCTGGGGCATCACT |
| iNOS | F: GTTCCAGGTGCACACAGGCT |
|  | R: GTGGTAGCCACATCCCGAGC |
| ARG1 | F: TCGGAACTCAACGGGAGGGT |
|  | R: TGCTTTGCTGTGATGCCCCA |
| TNF-α | F: GCCAACCAGGCAGGTTCTGT |
|  | R: TAGGCACCGCCTGGAGTTCT |
| TGF-β | F: AGGTCACCCGCGTGCTAATG |
|  | R: GCGTATCAGTGGGGGTCAGC |
| GAPDH | F: TGTGGATGGCCCCTCTGGAA |
|  | R: TGACCTTGCCCACAGCCTTG |
